# Evidence of Genetic Continuity in the Shortfin Mako Shark (*Isurus oxyrinchus*) Between the Eastern Atlantic and Mediterranean Sea

**DOI:** 10.1002/ece3.73261

**Published:** 2026-04-06

**Authors:** Gambardella Chiara, Giannelli Francesco, Fernandez‐Corredor Elena, García‐Barcelona Salvador, Jenrette Jeremy, Moro Stefano, Shea Brendan, Colloca Francesco, Romeo Teresa, Echwikhi Khaled, Zammit‐Chatti Maissa, Lemsi Chiheb, Ferretti Francesco, Trucchi Emiliano, Taboada Sergi, Navarro Joan

**Affiliations:** ^1^ Life and Environmental Sciences, Università Politecnica Delle Marche Ancona Italy; ^2^ Sicily Marine Center‐ BEOM Department, Stazione Zoologica Anton Dohrn Napoli Messina Sicily Italy; ^3^ Human Evolution Program, Department of Organismal Biology, Evolutionary Biology Centre Uppsala University Uppsala Sweden; ^4^ Center for the Human Past, Department of Organismal Biology Uppsala University Uppsala Sweden; ^5^ Institut de Ciències del Mar (ICM) CSIC Barcelona Spain; ^6^ Centro Oceanográfico de Málaga Instituto Español de Oceanografía (IEO‐CSIC) Málaga Spain; ^7^ Department of Fish and Wildlife Conservation, Virginia Tech Blacksburg Virginia USA; ^8^ Department of Integrative Marine Ecology, Stazione Zoologica Anton Dohrn Rome Italy; ^9^ Italian Institute for Environmental Protection and Research ISPRA Rome Italy; ^10^ Laboratory of Ecology and Environment, Faculty of Sciences of Gabès University of Gabes Gabes Tunisia; ^11^ Higher Institute of Biotechnology of Beja University of Jendouba Jendouba Tunisia; ^12^ Faculty of Sciences of Sfax University of Sfax Sfax Tunisia; ^13^ Department of Earth and Marine Sciences (DiSTeM) University of Palermo Palermo Italy; ^14^ Departamento de Biodiversidad y Biología Evolutiva Museo Nacional de Ciencias Naturales (MNCN), CSIC Madrid Spain

**Keywords:** connectivity, ddRAD, elasmobranch, lamnids, pelagic, population genetics

## Abstract

Highly migratory sharks such as the shortfin mako ( *Isurus oxyrinchus* ) undertake extensive oceanic movements that cross ecological and political boundaries. This wide‐ranging behavior increases their exposure to human impacts, primarily from fishing activities, as the species is frequently caught as bycatch in longline fisheries. Consequently, the shortfin mako is listed as Endangered globally and Critically Endangered in the Mediterranean Sea. Despite growing conservation concerns, knowledge of its population structure within the Mediterranean, and its genetic and ecological connectivity with the Atlantic Ocean, remains limited, hindering the development of effective management measures. We investigated the population structure and demographic history of shortfin mako across the Eastern Atlantic Ocean and Mediterranean Sea using double‐digest restriction‐site associated DNA (ddRADseq) sequencing on 66 individuals collected from six regions in the two areas. After sequence filtering, we retained 4349 neutral SNPs to assess population structure and demography. Our results indicated a single, genetically mixed population across the Eastern Atlantic‐Mediterranean range, suggesting high connectivity and gene flow. Demographic analyses revealed a historically stable population size, although recent declines may not be detectable given the resolution of our genetic markers. While such connectivity may enhance resilience to localized pressures, it also implies that intense Mediterranean fishing could have population‐wide consequences. These findings challenge the assumption of strong genetic isolation between basins but do not preclude demographic differentiation. Given that ICCAT currently treats the Mediterranean as a separate, data‐poor stock, our results highlight the need for coordinated, basin‐wide monitoring and management to safeguard this endangered species.

## Graphical abstract

Genomic analyses of shortfin mako sharks from the Eastern Atlantic and Mediterranean Sea reveal a single genetically mixed population, indicating high connectivity across basins. Demographic reconstruction suggests historical stability, though recent declines may be beyond the resolution of our markers. These results underscore the need for coordinated monitoring and management across both regions.

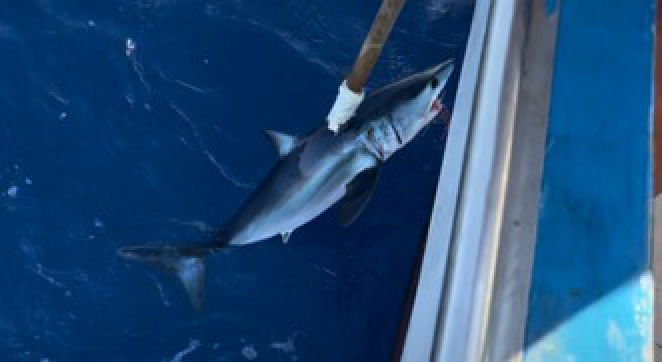

## 1 Introduction

Over the past few decades, large predatory sharks have experienced steep population declines across the world's oceans, triggering widespread concern for their conservation (Ferretti et al. 2010; Dulvy et al. 2017; Pacoureau et al. 2021). Apex predators, such as sharks, are involved in regulating prey populations and structuring trophic interactions, contributing to biodiversity and ecosystem stability (Estes et al. 2016; Birkmanis et al. 2020; Dedman et al. 2024). However, their life‐history traits, such as slow growth, late maturity, and low fecundity, make them particularly vulnerable to anthropogenic pressures, including overfishing and incidental bycatch (Lewison et al. 2004; Prato et al. 2013; Gallagher et al. 2014; Dulvy et al. 2021; Moro et al. 2025).

This vulnerability is particularly visible in oceanic and highly migratory sharks (Dulvy et al. 2008; Pacoureau et al. 2021), such as the shortfin mako ( *Isurus oxyrinchus* Rafinesque, 1810), which faces intense fishing pressure across its broad geographic range (Sims et al. 2021; Mucientes et al. 2025). Shortfin makos are the second most frequently caught shark species in global pelagic longline fisheries and are often retained in both commercial and recreational catches (Carpentieri et al. 2021; Bowlby et al. 2022). However, high overall capture levels do not necessarily translate into comprehensive or species‐specific datasets, particularly when captures occur predominantly as incidental bycatch. This pelagic shark is currently listed as Critically Endangered by the International Union for Conservation of Nature (IUCN) in the Mediterranean region and Endangered globally (Rigby et al. 2019), and is included under international management frameworks such as Convention on International Trade in Endangered Species of Wild Fauna and Flora (CITES), Convention on Migratory Species (CMS), and International Commission for the Conservation of Atlantic Tunas (ICCAT) (Fowler et al. 2021; ICCAT 2021). Despite these actions, the effectiveness of conservation efforts on shortfin mako sharks is hindered by persistent data gaps regarding their basic life history traits, population dynamics, connectivity, and demographic status (Camhi et al. 2007; Yokoi et al. 2017; Kai 2021; Fowler et al. 2021; Pacoureau et al. 2021).

Monitoring shortfin makos remains challenging due to their migratory nature, which implies crossing multiple marine exclusive economic zones and high‐seas regions, where monitoring and reporting frameworks differ substantially among jurisdictions, complicating both data collection and policy‐making (Dulvy et al. 2008; Yokoi et al. 2017; Corrigan et al. 2018; Mucientes et al. 2025). In addition, their low sighting rates in surveys (Cattano et al. 2023), limited surface residency (Gibson Banks et al. 2025), and the high cost of tracking technologies (Moses et al. 2022; Shea et al. 2024) have all contributed to their underrepresentation in ecological and fisheries datasets, especially in the Mediterranean Sea region (Dulvy et al. 2016; Cashion et al. 2019; Fernández‐Corredor et al. 2024). As a result, our understanding of their population structure remains incomplete and is largely inferred from indirect sources, such as bycatch records or tagging campaigns in other ocean basins, which are commonly used to define management stocks (Dulvy et al. 2016; Francis et al. 2018; González et al. 2023; Coelho et al. 2025).

In this context, genetic tools have provided valuable insights into the population structure and connectivity of wide‐ranging marine predators (e.g., Kool et al. 2013; Kacev 2015; González et al. 2023). For shortfin mako sharks, studies using mitochondrial DNA (mtDNA) revealed global‐scale genetic structure, suggesting regional differentiation potentially shaped by female philopatry (Heist et al. 1996; Taguchi et al. 2011; Vella and Vella 2023; González et al. 2023). However, subsequent analyses based on microsatellite loci, which are biparentally inherited and typically more variable, reported little to no differentiation across ocean basins, supporting widespread gene flow and genetic homogeneity on global scales (Schrey and Heist 2003). More recently, Corrigan et al. (2018) integrated mtDNA data with satellite telemetry and confirmed that while shortfin makos can move across ocean basins, their reproductive connectivity may be limited, particularly among females. These contrasting patterns between mitochondrial and nuclear markers underscore how different molecular markers may reveal contrasting aspects of connectivity (Corrigan et al. 2018; Domingues et al. 2022), and the importance of using higher‐resolution genomic tools to detect subtle population structuring, as it has been recently proved for a study using a population genomics approach in the blue shark (*Prionace glauca*, Linnaeus, 1758) (Leone et al. 2024). Similar patterns of homogeneity have also been reported in other large pelagic sharks, in which demographic separation is not related to genetic discontinuities (Bernard et al. 2016; Veríssimo et al. 2017; González et al. 2023; Leone et al. 2024). This has led to the recognition of a “population grey zone,” a demographic phase during which incomplete lineage sorting prevents clear identification of population units, even when ecological or behavioral differences are present (Bailleul et al. 2018). This notion is adapted from the “species grey zone” concept (De Queiroz 2007), in which lineages have diverged too recently to be fully distinguished genetically. In the population context, this lag between demographic separation and detectable genetic differentiation can lead to a misidentification of stock structure, particularly when genomic resolution available in previous studies is limited or sampling is opportunistic rather than a standardized sampling design (Bailleul et al. 2018). Blue sharks illustrate this pattern of displaying ecological heterogeneity while genetic studies continue to report little to no structure across ocean basins (Taguchi et al. 2013; Veríssimo et al. 2017; Bailleul et al. 2018; Leone et al. 2024). The shortfin mako, as another highly migratory oceanic shark, may similarly fall within this “*population grey zone*” concept. Although ecological or demographic isolation has been suggested across regions such as the Atlantic and Pacific oceans (Heist et al. 1996; Taguchi et al. 2011; Corrigan et al. 2018; Mehlrose 2022; González et al. 2023), nuclear DNA analyses have generally failed to detect strong genetic structure (Schrey and Heist 2003). Investigating whether this pattern extends to the Mediterranean is essential, as the region remains understudied under this genetic connectivity approach (ICCAT 2019).

The Mediterranean Sea is a semi‐enclosed and heavily exploited basin, where more than half of shark species are in decline (Dulvy et al. 2016, 2021). Yet it has been consistently excluded from global genetic studies of the shortfin mako (ICCAT 2021), despite the species being considered a separate stock in the Mediterranean Sea (ICCAT 2025). However, as of the latest ICCAT report (2025), data remain too limited to conduct a formal stock assessment. This gap is critical, as it reflects limitations in the availability and quality of biological and fisheries information required for formal stock assessment (European Commission et al. 2025), rather than low exploitation levels, particularly in a region characterized by ecological distinctiveness, intense anthropogenic pressures, and potential differences between Mediterranean and Atlantic populations (Patarnello et al. 2007; Reuschel et al. 2010).

To address this gap, we investigated the population structure and genetic diversity of shortfin mako sharks from the Mediterranean Sea and the Eastern Atlantic Ocean using double‐digest restriction site‐associated DNA sequencing (ddRADseq) (Peterson et al. 2012). This genome‐reduction approach enables the discovery of thousands of single nucleotide polymorphisms (SNPs) distributed across the genome, providing higher resolution than traditional mitochondrial or microsatellite markers previously used for this species. We aimed to assess whether genetic differentiation exists between the two areas and to investigate the ancient demographic history of the species (Domingues et al. 2022; Leone et al. 2024). This study provides the first population genomic analysis of shortfin mako sharks in the Mediterranean Sea and contributes essential data to implement spatially explicit, evidence‐based management strategies for this Critically Endangered pelagic predator.

## 2 Materials and Methods

### 2.1 Sample Collection

**FIGURE 1:**
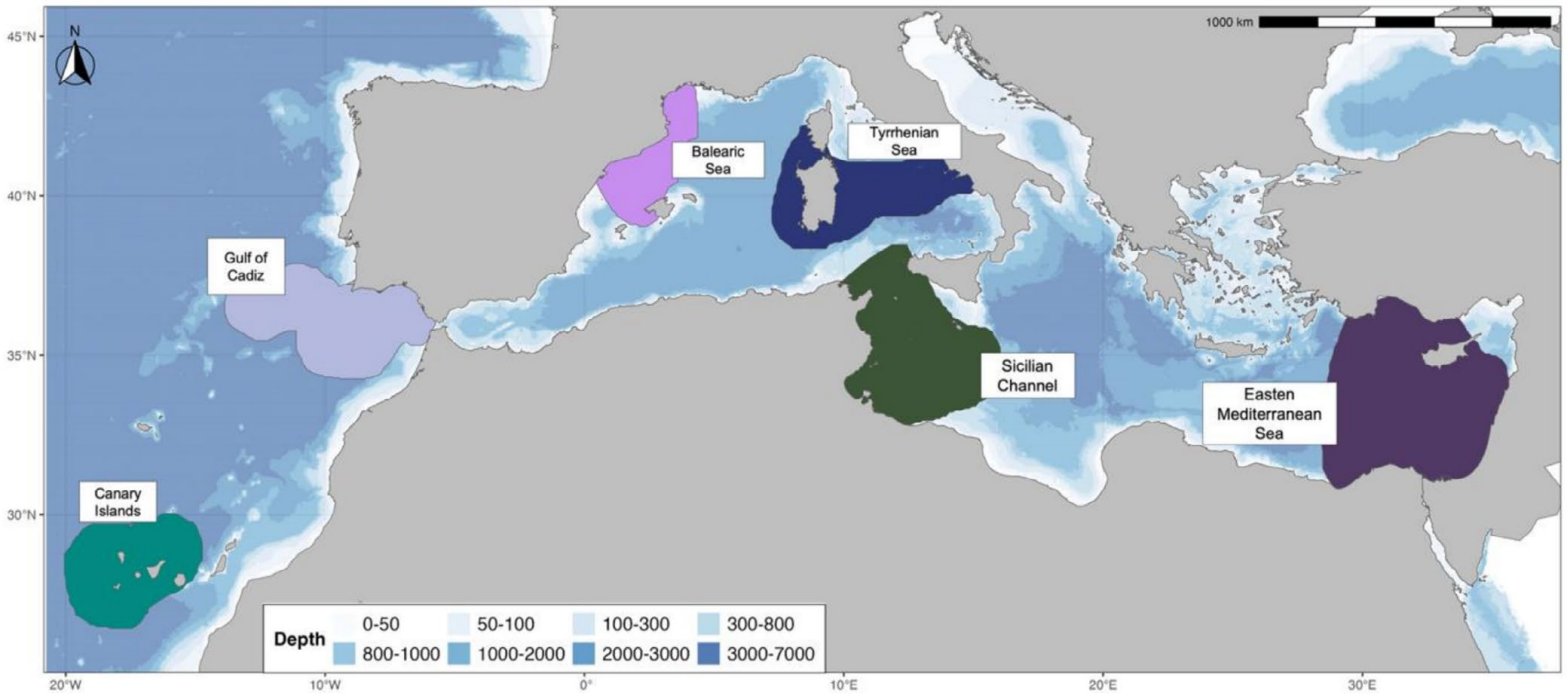
Map of the regions in which samples of shortfin mako sharks were collected (Gulf of Cadiz in light violet, Balearic Sea in pink, Canary Islands in emerald, Eastern Mediterranean in violet, Sicilian Channel in dark green, and Tyrrhenian Sea in dark blue). Bathymetric data of the area are also displayed (*source*: GEBCO https://download.gebco.net/).

A total of 81 samples of muscle and skin were collected from two regions in the Atlantic Ocean—the Gulf of Cadiz (GOC, *n* = 36) and Canary Islands (CAN, *n* = 11)—and from four regions in the Mediterranean Sea—Balearic Sea (BAL, *n* = 4), Tyrrhenian Sea (TYR, *n* = 3), Sicilian Channel (SIC, *n* = 23) and Eastern Mediterranean Sea (EMS, *n* = 4) (Figure 1, Table S1). All samples were collected from shortfin makos captured as bycatch from the longlining fishery in each sector. Muscle or skin tissue samples were collected using sterile scissors or tweezers and stored in 96% ethanol at −20°C until DNA extraction. Specimens' biological data, including fork length (in cm) and sex (female or male), as well as sampling data (e.g., catch date and location) were recorded whenever possible (Table S1). While fishery‐dependent bycatch sampling may introduce biases related to gear selectivity (e.g., size‐ and sex‐specific vulnerabilities in longline fisheries), this represents the primary opportunity to opportunistically collect samples from these wide‐ranging pelagic predators across large ocean basins, consistent with previous genetic studies of shortfin mako sharks and other elasmobranchs (Gilman et al. 2022; Baetscher et al. 2022; Scacco et al. 2023).

### 2.2 DNA Extraction, Library Preparation, and Sequencing

Genomic DNA (gDNA) was extracted from all samples using the DNeasy Blood & Tissue kit (Qiagen, www.qiagen.com) with slight adjustments to the manufacturer's protocol. Specifically, the cell lysis step was extended overnight, and the quantities of Proteinase K and lysis buffers were doubled through to the washing stage. DNA was eluted in a final volume of 80 μL using the provided elution buffer. Double‐stranded DNA concentration was measured using the Qubit dsDNA Broad Range Assay (Life Technologies).

Libraries for ddRADseq were produced for all samples following Peterson et al. (2012) with modifications following Combosch et al. (2017) at the Marine Molecular Lab Museo Nacional de Ciencias Naturales‐CSIC (Madrid, Spain). Double‐stranded gDNA (500 ng) was digested using the high‐fidelity restriction enzymes *EcoRI* and *SbfI* (New England Biolabs) for six hour at 37°C. The resulting digested fragments were cleaned by manual pipetting using Agencourt AMPure beads (1.5× volume ratio; Beckham Coulter) and were quantified with a Qubit dsDNA HS assay (Life Technologies). Fragmented DNA was ligated to custom P1 and P2 adapters, which included sample‐specific barcodes and PCR primer binding sites. Barcoded individuals were pooled into seven different libraries, cleaned by manual pipetting using AMPure beads (1.5× volume ratio), and size selected (range sizes 200–400 bp) using a Blue Pippin Prep (Sage Science). Each library was PCR‐amplified with Phusion polymerase (Thermo Scientific) using a different set of PCR primers, allowing for multiplexing libraries. The PCR program consisted of an initial denaturation at 98°C for 30 s, followed by 12 cycles of 98°C for 10 s, 65°C for 30 s, and 72°C for 90 s, and a final extension at 72°C for 10 min. The resulting PCR products were cleaned by manual pipetting using Agencourt AMPure beads (1.5× volume ratio) and then quantified with a Qubit dsDNA HS assay. The resulting products were also quality‐checked on a Tapestation 2200 (Agilent Technologies). Libraries were then pooled, normalizing their concentration, and they were pair‐end sequenced (2 × 150 bp) on an Illumina NovaSeq 6000 at Novogene Europe (Cambridge, UK).

### 2.3 Bioinformatics

High‐quality genomic DNA was successfully extracted from all individuals, and concentration and integrity were deemed sufficient for double‐digest restriction site‐associated DNA sequencing (ddRAD‐seq). Seven ddRAD libraries were prepared in total. Filtering and locus assembly were performed with the *Stacks* pipeline version 2.57 (Catchen et al. 2013; Paris et al. 2017). Initial demultiplexing and quality filtering were performed using the *process_radtags* module, which removed reads lacking complete barcodes or restriction sites, as well as low‐quality or ambiguous reads. To enhance recovery of slightly divergent barcodes and RAD‐tags, the rescue function (‐r) was applied with parameters *–barcode_dist 3* and *–adapter_mm 2*. All retained reads were trimmed to 140 bp using the trimming option (‐t) to ensure consistent read length and improve the accuracy of SNP detection.

After filtering and quality control, a total of 672,673,558 reads were retained from the initial 766,466,924 raw reads (representing approximately 87.8% of the original dataset). Of the initial 81 individuals across all libraries, two libraries (corresponding to Mako_L6 and Mako_L7) failed sequencing and were excluded due to extremely low read counts. The remaining five libraries (Mako_L1 to Mako_L5) passed quality control, demultiplexing, and trimming, with a total of 70 individuals.

The number of retained reads per individual ranged from 68,989 to 34,666,213. One individual (SC24‐9) with the lowest number of reads was excluded due to insufficient coverage, resulting in 69 individuals retained for downstream analysis. The lowest number of reads among retained individuals was 634,246 and the average number of reads per sample was 9,747,892.3, yielding a total of approximately 672,604,569 reads across the dataset. During initial exploratory analyses, three individuals formed a highly divergent cluster in different outputs, inconsistent with the rest of the dataset. Given their genetic distinctiveness and potential misidentification, they were excluded from the final analyses. The resulting filtered dataset comprised 66 shortfin mako shark individuals. The neutral SNPs were identified after filtering for missing data (≥ 80% genotyped), minor allele frequency (≥ 0.05), deviation from Hardy–Weinberg equilibrium (*p* < 0.05 in at least two populations), and removal of loci identified as outliers by both BayeScan and Arlequin. This final neutral dataset comprised 4349 high‐quality neutral SNPs (see Methods) with a mean locus length of 227.6 bp (±0.96) for 66 individuals.

Cleaned and filtered reads were then aligned to the shortfin mako scaffold‐level reference genome published by Stanhope et al. (2023) (Access number GCA_026770705.1 from NCBI) using a reference‐based assembly approach using BWA‐MEM (Li 2013). The reference genome was first indexed with BWA, and reads were aligned using default parameters. The resulting files were sorted and indexed using SAMtools (Danecek et al. 2021). The aligned reads were subsequently used as input for the *ref_map.pl* pipeline in *Stacks* to perform locus assembly and SNP calling.

To evaluate the effect of parameter choice, we conducted exploratory tests on a subset of individuals by varying adapter mismatch tolerance in the Stacks pipeline. These trials produced nearly identical results in terms of assembled loci and SNPs, so we proceeded with the parameter settings described.

We ran the Stacks *populations* module retaining loci present in at least 80% of all individuals (*r* = 0.8), and only the first SNP per RAD‐tag was retained (*–write‐single‐snp*) to reduce linkage disequilibrium. Additional filtering excluded loci with a minor allele frequency below 0.05 (*–min_maf 0.05*), loci significantly deviating from Hardy–Weinberg equilibrium (*p* < 0.05) in at least two populations, and loci with excess heterozygosity (Ho > 0.5). SNPs under selection were searched using *Arlequin* v 3.5.2.1 (Excoffier and Lischer 2010) and *Bayescan* v 2.1 (Foll and Gaggiotti 2008). *Arlequin* was run using “no hierarchical island model,” 100,000 simulations and 1000 demes per group; *p* values were corrected using the “*p.adjust*” function in R with the “*fdr*” method, corresponding to the “BH” in Benjamini and Hochberg (1995). *Bayescan* was run using a total of 10,000 output iterations and 100 prior odds; we considered outlier SNPs those with an FDR‐corrected *p* value (*q* value) > 0.05. Both approaches identified no SNPs under selection. This resulted in a dataset of 4349 neutral SNPs.

Patterns of missing data were assessed using the *adegenet* package (Jombart and Ahmed 2011) in R and the Matrix Condenser interface (Medeiros and Farrell 2018). For co‐ancestry inference with *fineRADstructure*, a separate dataset was generated without the *–write‐single‐snp* option (Malinsky et al. 2018). The software was run using the default settings (−x 100,000, −y 100,000, −z 1000 to assign individuals to populations, and −x 10,000 for the tree building), and the results were graphically interpreted using the *Finestructure* R package and the *fineRADstructurePlot. R* script, both included in the *fineRADstructure* software. The final dataset included all SNPs per locus, resulting in 31,326 SNPs.

### 2.4 Population Genomic and Demographic Analyses

We calculated genetic diversity and demographic statistics grouping samples in six different groups: GOC, CAN, BAL, TYR, SIC and EMS. Expected (He) and observed (Ho) heterozygosity were calculated per group and globally using *Stacks*. We assessed the population structure and genetic differentiation using different methods: *STRUCTURE* (Pritchard et al. 2000), the discriminant analysis of principal components (*DAPC*) as implemented in the *adegenet* v 2.2.10 R package (Jombart and Ahmed 2011), Principal Component Analysis (PCA) (Zheng et al. 2012), pairwise *F*_ST_ (Pembleton et al. 2013) and *fineRADstructure* (Malinsky et al. 2018).

We ran *STRUCTURE* with 200,000 MCMC iterations following a burn‐in of 100,000 iterations, using the admixture ancestry model and no sampling location as a prior, setting a putative *K* from 1 to 8 in a total of 15 independent iterations for each run (Falush et al. 2003). *CLUMPAK* (Kopelman et al. 2015) was used to summarize the replicates and visualize the structure plots.

DAPC analysis included the use of the function *snapclust.choose.k* to identify the most likely number of clusters based on Akaike Information Criterion (AIC) and Bayesian Information Criterion (BIC), using the *k*‐means algorithm (pop.ini = “*kmeans*”) with a maximum of 10 clusters (max = 10) and 100 iterations (max.iter = 100). Both AIC and BIC supported *K* = 1, indicating no detectable subdivision. To further explore whether subtle geographic structure could be visualized, we additionally performed a supervised DAPC by assigning individuals to their sampling area a priori. The number of retained principal components (PCs) was determined with cross‐validation using *xvalDapc* (100 replicates). Assignment probabilities were plotted with *assignplot*, and scatterplots of PCs were generated with *scatter.dapc* (Jombart et al. 2010). PCA was also conducted independently to visualize clustering of individuals without prior assumptions.

Pairwise *F*_ST_ and relative *p* values were computed following the Weir and Cockerham model (1984), and using the *StAMPP* R package (Pembleton et al. 2013). To assess if differentiation followed geographic pattern, we tested for isolation by distance (IBD) (Wright 1943) through a Mantel test (Mantel 1967), correlating pairwise *F*_ST_* = *F*_ST_/(1 − *F*_ST_) (Rousset 1997) with geographic distances among populations, as implemented in the *ade4* package in R.

Historical demographic trends were inferred using the *Stairway Plot* v2 (Liu and Fu 2020) on a filtered SNP dataset without the *–write‐single‐snp* option, retaining all SNPs per locus. We used the site frequency spectrum (SFS) constructed from all individuals treated as a single population. The folded SFS was employed to minimize potential bias from ancestral allele misidentification. The mutation rate was assumed to be 4.33e^−09^ following Stanhope et al. (2023) and the generation time was set to 18 years, corresponding to the reported age at female maturity for shortfin mako sharks (Natanson et al. 2006, 2020). Confidence intervals around effective population size estimates were obtained from 200 bootstrap replicates.

## 3 Results

Out of the 81 shortfin mako shark individuals collected, sex was determined for 26 specimens, of which 20 were female and 6 were male (Table S1). Fork length (FL) measurements were available for 72 individuals, ranging from 54.03 cm to 253.76 cm, with a mean of 119.59 cm (±40.23) (Table S1). Although sex and fork length data were available for a subset of individuals, the limited sample size across age classes and sexes prevented their inclusion in downstream genetic analyses.

Population genetic summary statistics revealed relatively homogeneous levels of genetic diversity across the six regions (Table 1). The number of private alleles varied across the regions, with the highest values observed in the GOC (925) and SIC (429), followed by CAN (261) (Table 1). By contrast, the BAL, EMS, and TYR had fewer private alleles (50, 57, and 64, respectively). When standardized by sample size, BAL (12.5) showed the lowest value, while all other regions showed values between 14 and 26 alleles per individual (Table 1).

**TABLE 1:** Summary of genetic indices for all individuals across Gulf of Cadiz, Balearic Sea, Canary Islands, Eastern Mediterranean, Sicilian Channel, Tyrrhenian Sea, and the overall values at the bottom.

Regions	*N*	Private alleles	Private alleles/*N*	Ho ± SD	He ± SD	π± sd
Gulf of Cadiz (GOC)	31	925	25.69	0.0778 ± 0.09	0.0852 ± 0.1	0.0867 ± 0.1
Balearic Sea (BAL)	4	50	12.50	0.075 ± 0.14	0.0726 ± 0.13	0.0841 ± 0.15
Canary Islands (CAN)	9	261	23.73	0.0763 ± 0.11	0.0814 ± 0.12	0.0865 ± 0.12
Eastern Mediterranean (EMS)	4	57	14.25	0.0545 ± 0.13	0.055 ± 0.12	0.0638 ± 0.14
Sicilian channel (SIC)	15	429	18.65	0.0776 ± 0.11	0.0843 ± 0.11	0.0874 ± 0.11
Tyrrhenian Sea (TYR)	3	64	21.33	0.0559 ± 0.13	0.0572 ± 0.13	0.0697 ± 0.15
OVERALL	66			0.1087 ± 0.0160	0.1151 ± 0.0181	0.1161 ± 0.0185

*Note:* Abbreviations for the different regions are given in brackets. Reported values include the number of individuals analyzed per region (*N*), number of Private alleles, standardized number of private alleles (Private alleles/*N*), mean observed heterozygosity (Ho) ± standard deviation (sd), mean expected heterozygosity (He) ± sd, mean nucleotide diversity (*π*) ± sd.

When considering all individuals together (Table 1, *N* = 66), observed heterozygosity (Ho) was 0.1087 ± 0.0160, expected heterozygosity (He) was 0.1151 ± 0.0181, and nucleotide diversity (π) was 0.1161 ± 0.0185 (Figure S1). Regionally, Ho ranged from 0.055 (EMS and TYR) to 0.078 (CAN), while He ranged from 0.057 (EMS) to 0.085 (GOC). Nucleotide diversity (π) followed a similar pattern, with the lowest values in TYR (π= 0.064) and EMS (π = 0.064), and the highest in SIC (π = 0.087) and GOC (π = 0.087) (Table 1).

**FIGURE 2:**
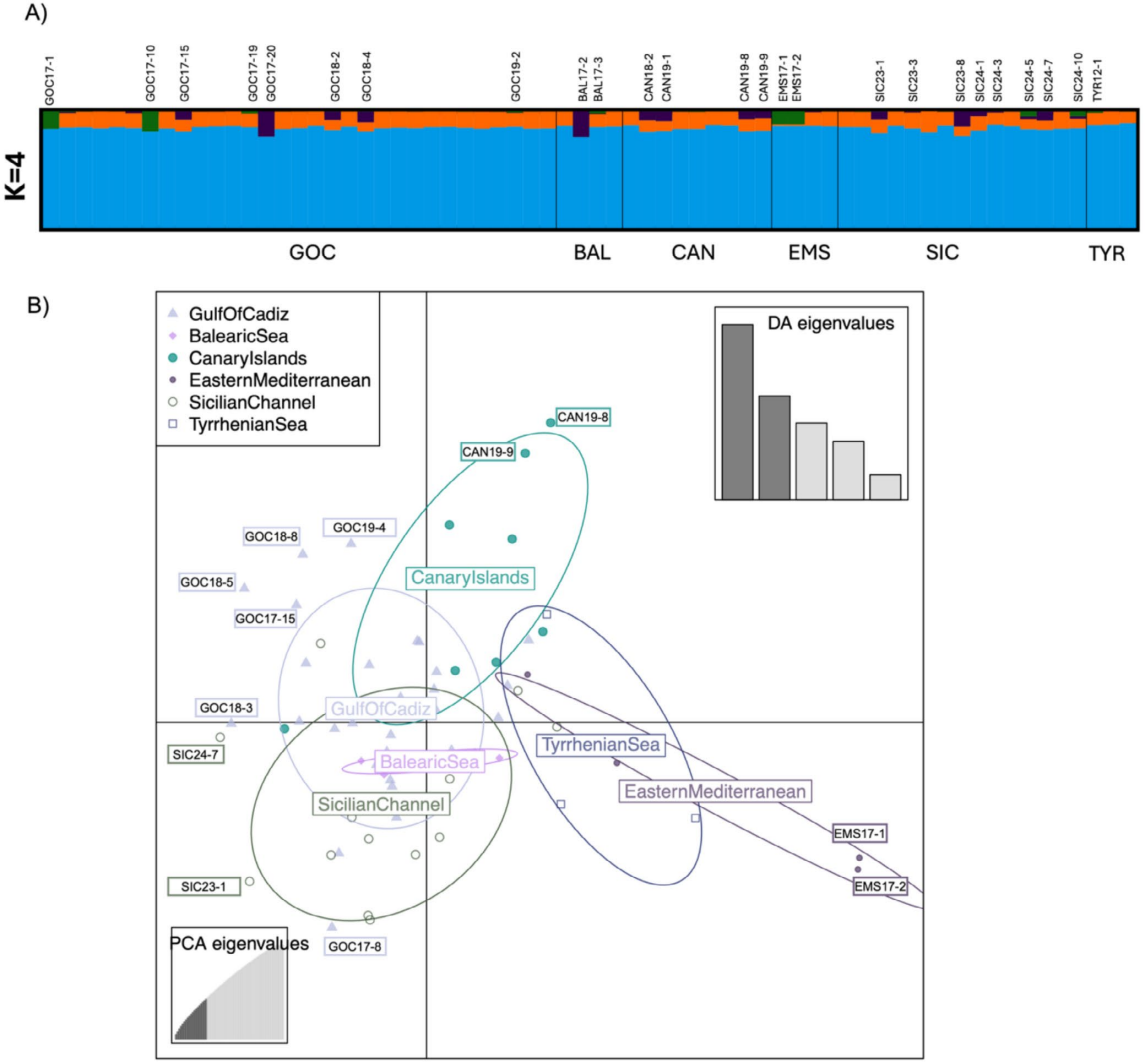
(A) Bayesian clustering analysis of shortfin mako sharks using STRUCTURE. Each vertical bar represents an individual, and colors indicate inferred ancestry proportions from *K* genetic clusters. Individuals are grouped by sampling region: Gulf of Cadiz (GOC), Balearic Sea (BAL), Canary Islands (CAN), Eastern Mediterranean (EMS), Sicilian Channel (SIC), and Tyrrhenian Sea (TYR). Results are shown for *K* = 4. Individuals showing a genetic assignment to the purple and green clusters are highlighted, (B) Discriminant Analysis of Principal Components (DAPC) of 66 shortfin mako sharks (4349 neutral SNPs) grouping the samples by region. Individuals are represented as points, colored and shaped according to sampling region (Gulf of Cadiz in light violet, Balearic in pink, Canary Islands in emerald, Eastern Mediterranean in violet, Sicilian Channel in dark green, and Tyrrhenian Sea in dark blue). Ellipses represent 95% confidence intervals around group centroids. Labels indicate the individuals most distant from their region centroids in the DAPC space. These partially overlap with, but are not identical to, the individuals highlighted by STRUCTURE in panel A, reflecting that DAPC captures between‐group dispersion whereas STRUCTURE infers admixture proportions. Insets show the proportion of variance explained by retained PCA eigenvalues (bottom left) and the discriminant analysis eigenvalues (bottom right), which illustrate the relative contribution of each discriminant function to group separation.

The STRUCTURE analyses identified *K* = 4 as the most likely number of clusters according to the Evanno Δ*K* method (Figure 2A, Figure S2), with all the individuals being primarily assigned to the blue cluster (> 80% genetic assignment) and most of them showing a varying degree of assignation to the orange cluster; a few individuals showed instead a secondary assignment to the purple and green clusters (Figure 2A). The genetic assignment did not correspond to geographic sampling regions but detected a single panmictic (i.e., individuals breed randomly within a single gene pool) population with no genetic structure (Figure 2A). Consistently, model selection criteria (AIC and BIC) from the DAPC analysis supported *K* = 1 as the best option (Figure S3). When higher values of *K* were forced (*K* = 2:4; Figure 2B and Figure S4), individuals were artificially split into clusters, but these partitions showed strong overlap, lacked geographic consistency, and were not always reproducible across methods. Few individuals fell outside the DAPC ellipses, partially overlapping with those highlighted by STRUCTURE at *K* = 4 (Figure 2A,B). This pattern reflects that DAPC emphasizes between‐group dispersion, whereas STRUCTURE infers admixture proportions. For example, a few individuals from the Eastern Mediterranean and Canary Islands appeared partially separated from the main cluster (Figure 2B), but this signal was not consistent. Similarly, PCA revealed no clear geographic clustering, with individuals from all regions broadly overlapping when comparing PC1 vs. PC2 and PC2 vs. PC3 (Figure S5). The first axis (PC1) explained 3.75% of the variance and the second (PC2) 2.6%, with all regions broadly overlapping (Figure S5). The projection of PC2 against PC3 (2.6% and 2.2% of variance, respectively) showed a similar lack of structure, although a few individuals (e.g., SIC23‐6, GOC17‐1, GOC17‐10) appeared slightly separated from the main cluster (Figure S5). These scattered individuals were not consistently differentiated across other analyses, confirming the overall panmictic signal.

**FIGURE 3:**
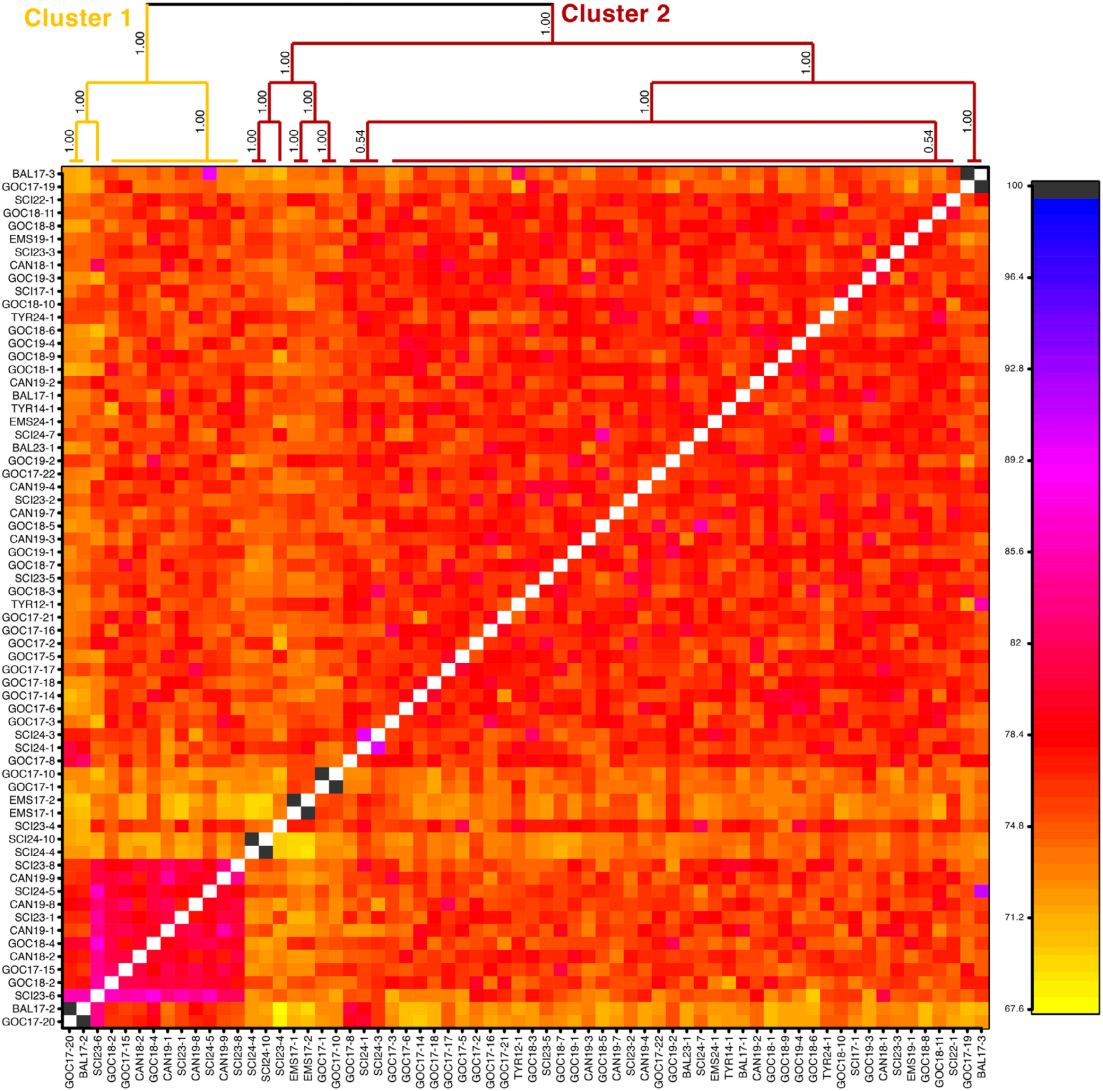
Co‐ancestry heatmap generated with fineRADstructure based on SNP data from 66 shortfin mako individuals. Each cell represents the proportion of shared genetic ancestry between pairs of individuals, with warmer colors (yellow–red) indicating lower co‐ancestry and cooler colors (pink–blue) indicating higher co‐ancestry. The hierarchical tree above the heatmap identifies two clusters: Cluster 1, including most individuals from all sampling regions, and Cluster 2, grouping a subset of individuals (notably SCI23‐8 and SCI23‐1 from the Sicilian Channel, GOC17‐20 and GOC17‐15 from the Gulf of Cadiz, together with several others). Many of the same individuals also showed mixed ancestry in the STRUCTURE analysis (Figure 2A), confirming subtle differentiation without a geographic basis. Overall, the analysis highlights high connectivity across the dataset.

**FIGURE 4:**
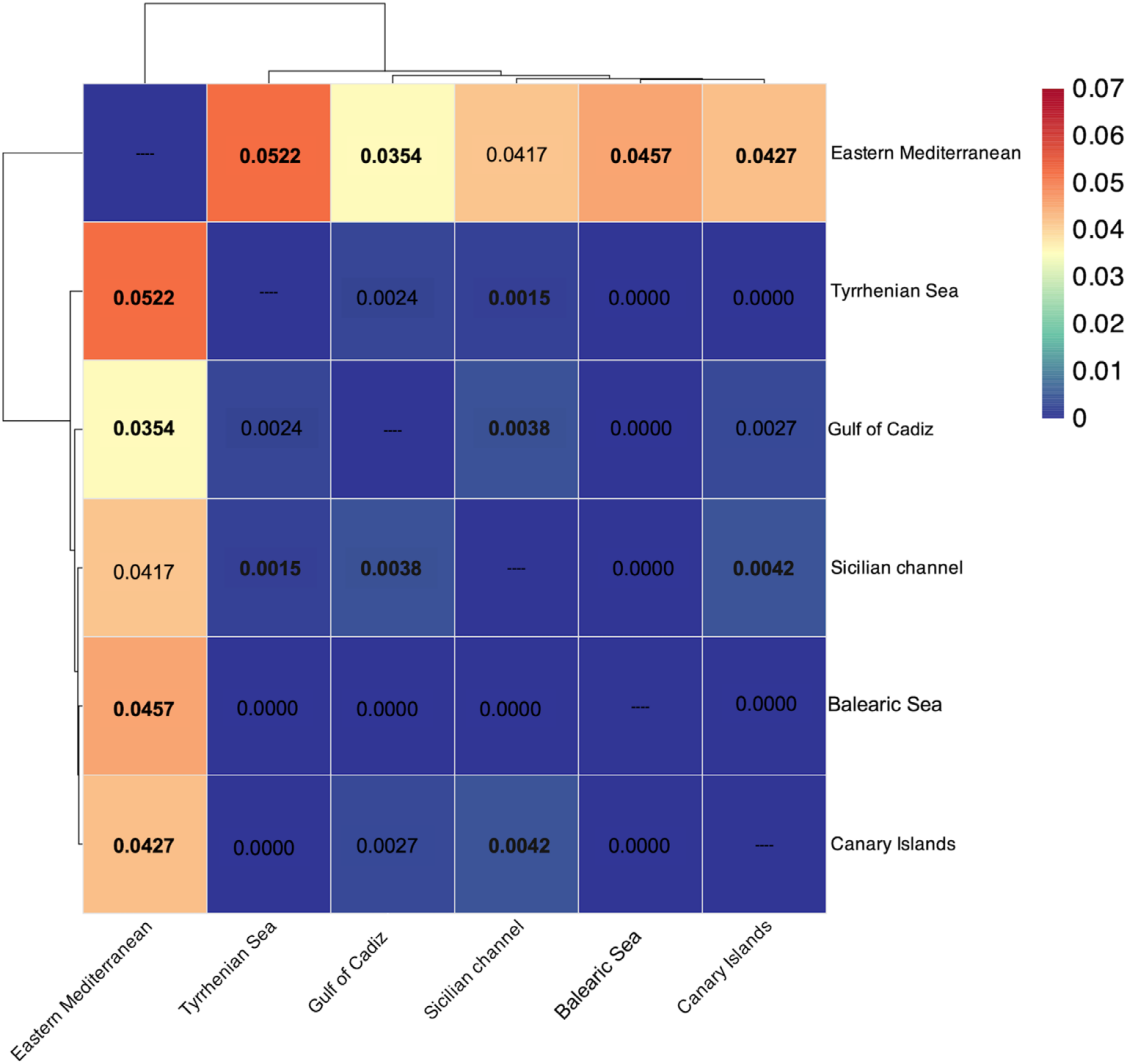
Pairwise genetic differentiation (FST) among shortfin mako sharks from six geographic regions: Gulf of Cadiz, Balearic Sea, Canary Islands, Eastern Mediterranean, Sicilian Channel, and Tyrrhenian Sea. Colors represent the magnitude of FST values (scale on the right), with higher values indicating stronger differentiation. Numbers in the cells are pairwise FST estimates; significant comparisons (*p* < 0.05 after correction) are shown in bold; clustering dendrograms are based on hierarchical clustering of the FST matrix. The plot highlights the distinct differentiation of the Eastern Mediterranean compared with the other regions, which show very low FST values.

The *FineRADstructure* co‐ancestry matrix showed high levels of shared ancestry across all individuals, with subtle evidence of distinct clusters (Cluster 1 and Cluster 2, Figure 3). Cluster 1 included individuals from all sampling regions, whereas Cluster 2 grouped a small subset of individuals (notably SIC23‐8 and SIC23‐1 from the Sicilian Channel, GOC17‐20 and GOC17‐15 from the Gulf of Cadiz, together with several others) most of which corresponded to individuals assigned to the purple cluster in STRUCTURE at *K* = 4 (Figure 2A). Nevertheless, these individuals were not consistently distinguished in other analyses, and both clusters contained sharks from the Eastern Atlantic Ocean and Mediterranean, indicating no geographic separation. Pairwise *F*_*ST*_ estimates revealed low genetic differentiation among regions (Figure 4). The highest levels of differentiation were observed between the EMS and all other regions, with *F*_*ST*_ values ranging from 0.035 to 0.052 (all *p* < 0.001). Significant values were also found between the SIC and both the CAN (*F*_*ST*_ = 0.004, *p* = 0.039) and the GOC (*F*_*ST*_ = 0.004, *p* < 0.001) (Figure 4). The GOC and CAN comparison resulted in a statistically significant *F*_*ST*_ (*p* < 0.05), though the low value (0.003) still indicates minimal differentiation. All other comparisons, including those involving the BAL and the TYR, showed nonsignificant *F*_*ST*_ values close to zero (Figure 4). No pattern of isolation by distance was detected among populations (Mantel test, *p* > 0.05) (Figure S6).

**FIGURE 5:**
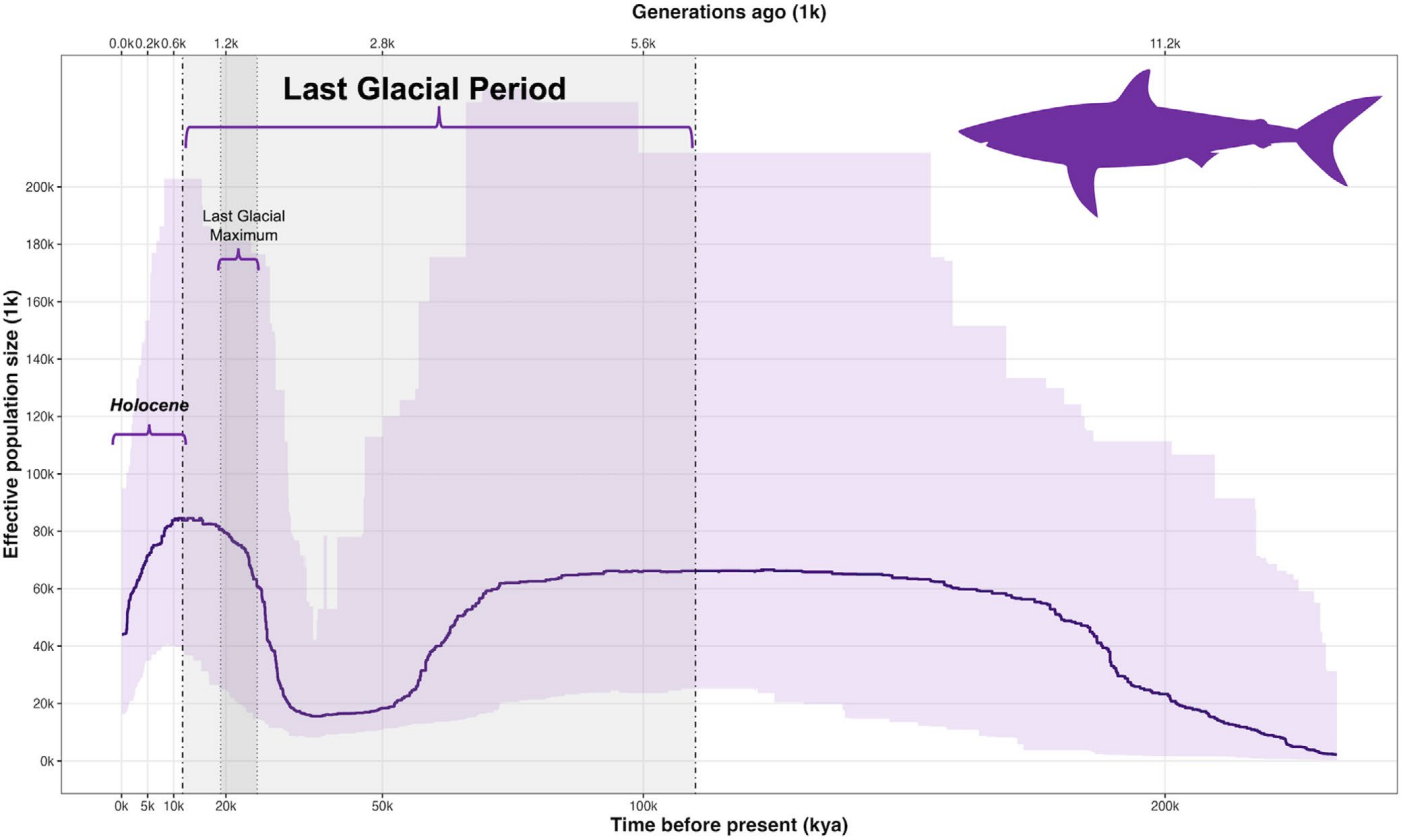
Historical demographic reconstruction of shortfin mako sharks based on the site frequency spectrum using Stairway Plot v2. The solid line represents the inferred effective population size (Ne) through time, and the shaded area indicates 95% confidence intervals estimated from 200 bootstrap replicates. Time is shown in thousands of years before present (kya), with vertical dashed lines and curly brackets marking the Last Glacial Period (LGP, ~11–110 kya), the Last Glacial Maximum (LGM, ~19–26 kya) and the Holocene (~11–today). The analysis assumed a mutation rate of 4.33 × 10–9 (Stanhope et al. 2023) and a generation time of 18 years (Natanson et al. 2006, 2020).

Demographic inference using Stairway Plot revealed a signal of a stable historical population followed by a contraction in effective population size, which likely occurred during the Last Glacial Period (around 11–110 kya, Behre 1989; Pascual et al. 2017) (Figure 5). The mean nucleotide diversity estimated from this dataset was 0.22842.

## 4 Discussion

Our study represents the first genome‐wide analysis of the shortfin mako shark including samples from the Mediterranean Sea. We generated a dataset of 4349 neutral SNPs from 66 individuals sampled across four regions in the Mediterranean and two regions in the Eastern Atlantic Ocean. Genetic diversity was relatively homogeneous among regions, with similar levels of heterozygosity and nucleotide diversity, except for a weak and statistically significant *F*_*ST*_ value between the Eastern Mediterranean and all other regions. Clustering analyses (STRUCTURE, DAPC, PCA, *fineRADstructure*) confirmed broad overlap among individuals, and no pattern of isolation by distance was detected. Historical demographic inference further suggested long‐term population stability followed by a contraction around the Last Glacial Period.

Overall, our findings align with previous work on the population structure of the shortfin mako shark carried out in the Atlantic and Pacific Oceans, which reported either weak or no genetic differentiation across broad geographic areas, particularly when using nuclear markers such as microsatellites or SNPs (Schrey and Heist 2003; Corrigan et al. 2018). On the other hand, studies based on mtDNA suggested global phylogeographic structure with significant differentiation across hemispheres and ocean basins, likely driven by female philopatry (Heist et al. 1996; Taguchi et al. 2011; Corrigan et al. 2018; González et al. 2023). This mitonuclear discordance is a well‐documented phenomenon in marine taxa and it is often interpreted as evidence of sex‐biased dispersal, where female philopatry contrasts with male‐mediated gene flow (Toews and Brelsford 2012). The only previous genetic study on the shortfin mako, which focused specifically on the Mediterranean Sea (Vella and Vella 2023), used mtDNA control region sequences. It found some unique haplotypes and a slightly lower haplotype diversity compared to other regions, but no significant divergence from the adjacent northeastern Atlantic Ocean.

The *F*_*ST*_ values we obtained in the Eastern Mediterranean should be interpreted with caution, since the number of samples for the different regions was unbalanced and in some cases the sample size is small, thus limiting the robustness of the comparisons (Nazareno et al. 2017; McLaughlin and Winker 2020). Nonetheless, this area is understudied, with indications of distinct and possibly underreported fishing pressures (Marsaglia et al. 2025) and different environmental conditions compared to the western Mediterranean and Atlantic Ocean (Papaconstantinou and Farrugio 2000; Cardinale et al. 2017; Demirel et al. 2020), which may explain the subtle degree of demographic separation detected. Our clustering analyses supported connectivity, further confirmed by the absence of isolation by distance. This suggests that gene flow occurs across broad spatial scales, rather than declining gradually with geographic distance (Mantel test *p* > 0.05) (Veríssimo et al. 2017; Corrigan et al. 2018; Domingues et al. 2018). Comparable patterns have been observed in blue sharks and other large pelagic fishes, where subtle structure may exist but remains challenging to detect with confidence given both biological traits and methodological constraints associated with studying open‐ocean species (Chapman et al. 2009; Veríssimo et al. 2017; Antoniou et al. 2017; González et al. 2023; Leone et al. 2024). These results revealing panmictic populations at large scales are common in wide‐ranging marine predators (Pascual et al. 2017), where apparent clustering may reflect local abundance or the earliest stages of demographic separation rather than true isolation (Palumbi 1994; Patarnello et al. 2007; Olsen et al. 2014; Bailleul et al. 2018; Corrigan et al. 2018; Domingues et al. 2018).

Our findings may fit with the “population grey zone” theory (Bailleul et al. 2018), a phase in which demographic separation has begun but genetic signals remain too weak to be consistently detected by conventional population genetics tools, because gene flow is still occurring at rates sufficient to homogenize allele frequencies (De Queiroz 2007; Bailleul et al. 2018). In the case of the shortfin mako shark, such separation could be driven by strong and regionally variable fishing pressure, particularly longline bycatch in the Mediterranean Sea, where high exploitation rates and underreporting create data gaps that limit stock assessments (Colloca et al. 2017; Lazaris et al. 2025; ICCAT 2025). Associated population declines over the past century (Ferretti et al. 2008; ICCAT 2019; Sims et al. 2021; Milazzo et al. 2021) may selectively deplete vulnerable age classes such as juveniles and gravid females (Maia et al. 2007; Scacco et al. 2023), contributing to demographic vulnerability and potentially to the subtle Eastern Mediterranean genetic signal observed here despite ongoing connectivity. Nevertheless, for species with high dispersal ability and long generation times, such as the shortfin mako (Corrigan et al. 2018; Natanson et al. 2020), this lag between demographic isolation and the appearance of measurable genetic divergence can span thousands of years (Bailleul et al. 2018). This makes it challenging to delineate distinct stocks solely based on genetic criteria and emphasizes the need to integrate genetic data with telemetry and ecological information (Domingues et al. 2018, 2021; Dedman et al. 2024).

Tagging studies support evidence of extensive movements of shortfin mako sharks across oceanic regions, including evidence of connectivity between the western and eastern Atlantic (Queiroz et al. 2019; Santos et al. 2021; Mucientes et al. 2025; Coelho et al. 2025). However, direct evidence of individuals entering the Mediterranean from the Atlantic remains scarce or anecdotal (Santos et al. 2021; Shea et al. 2024). The apparent absence of crossings through the Strait of Gibraltar in telemetry datasets may be due to small sample sizes or the rarity of such events, rather than a true barrier to gene flow. This pattern mirrors observations in blue sharks, which exhibit high mobility and apparent panmixia across ocean basins despite heterogeneity of habitats and evidence of partial reproductive isolation (Patarnello et al. 2007; Veríssimo et al. 2017; Bailleul et al. 2018; Santos et al. 2021; Leone et al. 2024).

From a methodological standpoint, our study represents the first use of ddRADseq to assess the population structure of shortfin mako sharks in the Mediterranean Sea, adding valuable resolution compared to earlier approaches based on mtDNA or microsatellites (Corrigan et al. 2018; Vella and Vella 2023).

Understanding demographic history is essential to interpret how past changes have shaped the current distribution and genetic diversity of marine species (Banks et al. 2013; Stoffel et al. 2018). Our demographic analyses suggested a long‐term population stability followed by a contraction happening in correspondence with the Last Glacial Period (Pascual et al. 2017; Stanhope et al. 2023). The Late Pleistocene fluctuations we highlighted is consistent with large‐scale environmental shifts that would plausibly change suitable pelagic habitat and prey availability (Six et al. 2024). During the Last Glacial Period/Last Glacial Maximum (110 Kya—11 Kya years before present), the Mediterranean experienced cooler surface temperatures and a ~90–130 m sea‐level fall (Bianchi et al. 2011), and a reshaping of circulation and exchange at Gibraltar (Bianchi et al. 2011). Moreover, coupled biogeochemical modeling indicated reduced net primary production and stronger water‐column stability, meaning more stratified waters with weaker vertical mixing compared to the Holocene (Bianchi et al. 2011). These features together would reduce foraging opportunities and nursery habitat for highly migratory pelagic species such as the shortfin mako, producing the contraction we infer. Comparable Late Pleistocene contractions (often followed by postglacial expansion) are reported for other shark species (Spaet et al. 2015), suggesting a basin‐wide climate imprint rather than a species‐specific anomaly. However, allele‐frequency–based inferences like the Stairway Plot have limited resolution for very recent (century‐scale) declines (Liu and Fu 2020). So, contemporary fishery‐driven declines (Ferretti et al. 2008; Milazzo et al. 2021; Cattano et al. 2023; Shea et al. 2024) may not yet have left detectable signatures in the genome, especially given the resolution of the data and the time lag required for demographic events to generate measurable genetic signals. This highlights once again the risk of assuming demographic unity based on genetic panmixia, especially for exploited species with high dispersal ability (Veríssimo et al. 2017; Corrigan et al. 2018; Bailleul et al. 2018).

ICCAT currently recognizes two shortfin mako management stocks in the Atlantic Ocean: North Atlantic (SMA‐N) and South Atlantic (SMA‐S), and a separate management stock for the Mediterranean Sea (SMA‐MED). However, the Mediterranean stock remains data‐poor and has never been quantitatively assessed (ICCAT 2021, 2025). Evidence of migration between the Eastern Atlantic and Mediterranean is limited to genetic signals of panmixia. The 2025 ICCAT Data Preparatory Meeting reiterated that information for the Mediterranean is still insufficient for a traditional stock assessment and emphasized the urgent need to define stock boundaries and improve regional data collection.

Our genomic data reveal connectivity between the Mediterranean and eastern Atlantic, indicating that individuals from different regions interbreed and share alleles. However, genetic connectivity does not imply demographic homogeneity, as it provides no information on local abundance, recruitment, or mortality rates (Hedgecock et al. 2007; Lowe and Allendorf 2010). Distinct environmental conditions and potentially underreported fishing pressures in the Mediterranean (Papaconstantinou and Farrugio 2000; Cardinale et al. 2017; Demirel et al. 2020; Marsaglia et al. 2025) may result in differential population dynamics despite shared ancestry. The shortfin mako is one of the most heavily fished sharks in the Atlantic and Mediterranean, facing high mortality rates and insufficient protection (ICCAT 2021; Pacoureau et al. 2023). Effective management requires integrating genomic evidence with ecological data and fisheries information, including fishing effort, bycatch rates, size‐ and sex composition of catches, and post‐release mortality to clarify population boundaries and identify region‐specific vulnerabilities (Domingues et al. 2018).

We propose that Mediterranean shortfin mako sharks should be considered part of a broader Atlantic‐Mediterranean genetic population yet managed as a demographically distinct unit exposed to unique regional pressures (Dulvy et al. 2016; Yokoi et al. 2017; Domingues et al. 2018; Milazzo et al. 2021; Dedman et al. 2024). Future research should aim to combine high‐resolution genomic tools, such as whole genome resequencing or targeted SNP panels, with biologically informed sampling strategies and complementary data from tagging and fisheries records. Such integrative approaches are essential to move beyond binary models of structure versus panmixia and toward a more nuanced understanding of connectivity in pelagic ecosystems.

## Author Contributions

**Gambardella Chiara:** conceptualization (lead), data curation (equal), formal analysis (equal), visualization (lead), writing – original draft (lead). **Giannelli Francesco:** formal analysis (supporting), writing – review and editing (equal). **Fernandez‐Corredor Elena:** writing – review and editing (equal). **García‐Barcelona Salvador:** resources (equal). **Jenrette Jeremy:** writing – review and editing (equal). **Moro Stefano:** writing – review and editing (equal). **Shea Brendan:** writing – review and editing (equal). **Colloca Francesco:** writing – review and editing (equal). **Romeo Teresa:** writing – review and editing (equal). **Echwikhi Khaled:** resources (equal). **Zammit‐Chatti Maissa:** resources (equal). **Lemsi Chiheb:** resources (equal). **Ferretti Francesco:** project administration (equal), writing – review and editing (equal). **Trucchi Emiliano:** methodology (supporting), supervision (supporting), writing – review and editing (equal). **Taboada Sergi:** data curation (equal), formal analysis (supporting), methodology (equal), supervision (equal), writing – review and editing (equal). **Navarro Joan:** funding acquisition (equal), project administration (equal), resources (equal), writing – review and editing (supporting).

## Funding

This study is part of the PhD thesis of CG and is an output of the project COTI funded by Fundación Biodiversidad and the Ministerio para la Transición Ecológica y el Reto Demográfico through the PLEAMAR Programme and co‐financed by the European Union through the FEMPA. Part of the sampling has been funded by the EU through the European Maritime and Fisheries and Aquaculture Fund (EMFAF) within the National Program for the management and use of data from the fisheries sector and support for scientific advice on the Common Fisheries Policy. ST received funding from the grants PID2020‐117115GA‐I00 and PID2024‐159957NB‐I00 funded by MCIN/AEI/10.13039/501100011033 and from the grant CNS2023‐144572 and by the Ramón y Cajal grant RYC2021‐03152‐I, funded by the MCIN/AEI/10.13039/501100011033 and the European Union «NextGenerationEU»/PRTR. We acknowledge the Atlantic White Shark Conservancy for supporting the sampling in the Sicilian Channel. Open access publication fees were covered through an APC voucher provided by Università Politecnica delle Marche.

## Disclosure

Benefit‐sharing statement: Benefits from this research accrue from the sharing of our data and results on public databases as described above.

## Ethics Statement

Samples were collected under the SGBTM/BDM/AUTSPP/76 permit, with no ethics approval required.

## Conflicts of Interest

The authors declare no conflicts of interest.

## Supporting information


**Data S1:** ece373261‐sup‐0001‐Supinfo.pdf.

## Data Availability

Raw ddRAD‐seq reads for the shortfin mako shark ( *Isurus oxyrinchus* ) have been deposited in the NCBI Sequence Read Archive (SRA) under BioProject accession PRJNA1359587. Individual BioSamples are available under accessions SAMN53172667–SAMN53172736. Metadata for each sample are present in the Supporting Information.

## References

[ece373261-bib-0001] Antoniou, A. , P. Kasapidis , G. Kotoulas , C. C. Mylonas , and A. Magoulas . 2017. “Genetic Diversity of Atlantic Bluefin Tuna in the Mediterranean Sea: Insights From Genome‐Wide SNPs and Microsatellites.” Journal of Biological Research‐Thessaloniki 24: 3. 10.1186/s40709-017-0062-2. PMC5314471 28239596

[ece373261-bib-0002] Baetscher, D. S. , J. Beck , E. C. Anderson , et al. 2022. “Genetic Assignment of Fisheries Bycatch Reveals Disproportionate Mortality Among Alaska Northern Fulmar Breeding Colonies.” Evolutionary Applications 15: 447–458. 10.1111/eva.13357. 35386403 PMC8965376

[ece373261-bib-0003] Bailleul, D. , A. Mackenzie , O. Sacchi , F. Poisson , N. Bierne , and S. Arnaud‐Haond . 2018. “Large‐Scale Genetic Panmixia in the Blue Shark ( *Prionace glauca* ): A Single Worldwide Population, or a Genetic Lag‐Time Effect of the “Grey Zone” of Differentiation?” Evolutionary Applications 11: 614–630. 10.1111/eva.12591. 29875806 PMC5978958

[ece373261-bib-0004] Banks, S. C. , G. J. Cary , A. L. Smith , et al. 2013. “How Does Ecological Disturbance Influence Genetic Diversity?” Trends in Ecology & Evolution 28: 670–679. 10.1016/j.tree.2013.08.005. 24054910

[ece373261-bib-0005] Behre, K.‐E. 1989. “Biostratigraphy of the Last Glacial Period in Europe.” Quaternary Science Reviews 8: 25–44. 10.1016/0277-3791(89)90019-X.

[ece373261-bib-0111] Benjamini, Y. , and Y. Hochberg . 1995. “Controlling the False Discovery Rate: A Practical and Powerful Approach to Multiple Testing.” Journal of the Royal statistical society: series B (Methodological) 57: 289–300. 10.1111/j.2517-6161.1995.tb02031.x.

[ece373261-bib-0006] Bernard, A. M. , K. A. Feldheim , M. R. Heithaus , S. P. Wintner , B. M. Wetherbee , and M. S. Shivji . 2016. “Global Population Genetic Dynamics of a Highly Migratory, Apex Predator Shark.” Molecular Ecology 25: 5312–5329. 10.1111/mec.13845. 27662523

[ece373261-bib-0007] Bianchi, C. N. , C. Morri , M. Chiantore , M. Montefalcone , V. Parravicini , and A. Rovere . 2011. “Mediterranean Sea Biodiversity Between the Legacy From the Past and a Future of Change.” In Life in the Mediterranean Sea: A Look at Habitat Changes, edited by N. Stambler , Nova Publisher.

[ece373261-bib-0008] Birkmanis, C. A. , J. C. Partridge , L. W. Simmons , M. R. Heupel , and A. M. M. Sequeira . 2020. “Shark Conservation Hindered by Lack of Habitat Protection.” Global Ecology and Conservation 21: e00862. 10.1016/j.gecco.2019.e00862.

[ece373261-bib-0009] Bowlby, H. D. , J. C. Peterson , W. N. Joyce , M. R. Simpson , and Oceans Canada, H D Bowlby . 2022. “Recovery Potential Assessment for the North Atlantic Designatable Unit of Shortfin Mako Shark (*Isurus oxyrinchus*).” 10.13140/RG.2.2.15007.78242.

[ece373261-bib-0010] Camhi, M. D. , S. Valenti , S. V. Fordham , S. L. Fowler , and C. Gibson . 2007. “The Conservation Status of Pelagic Sharks and Rays.” IUCN Shark Specialist Group Pelagic Shark Red List Workshop. Tubney House, University of Oxford, UK.

[ece373261-bib-0011] Cardinale, M. , G. C. Osio , and G. Scarcella . 2017. “Mediterranean Sea: A Failure of the European Fisheries Management System.” Frontiers in Marine Science 4: 72. 10.3389/fmars.2017.00072.

[ece373261-bib-0012] Carpentieri, P. , A. Nastasi , M. Sessa , and A. Srour . 2021. “Incidental Catch of Vulnerable Species in Mediterranean and Black Sea Fisheries–A Review.” (FAO). 10.4060/cb5405en.

[ece373261-bib-0013] Cashion, M. , N. Bailly , and D. Pauly . 2019. “Official Catch Data Underrepresent Shark and Ray Taxa Caught in Mediterranean and Black Sea Fisheries.” Marine Policy 105: 1–9. 10.1016/j.marpol.2019.02.041.

[ece373261-bib-0014] Catchen, J. , P. A. Hohenlohe , S. Bassham , A. Amores , and W. A. Cresko . 2013. “Stacks: An Analysis Tool Set for Population Genomics.” Molecular Ecology 22: 3124–3140. 10.1111/mec.12354. 23701397 PMC3936987

[ece373261-bib-0015] Cattano, C. , C. Gambardella , D. Grancagnolo , et al. 2023. “Multiple Interannual Records of Young‐Of‐The‐Year Identify an Important Area for the Protection of the Shortfin Mako, *Isurus oxyrinchus* .” Marine Environmental Research 192: 106217. 10.1016/j.marenvres.2023.106217. 37866201

[ece373261-bib-0016] Chapman, D. D. , D. Pinhal , and M. S. Shivji . 2009. “Tracking the Fin Trade: Genetic Stock Identification in Western Atlantic Scalloped Hammerhead Sharks *Sphyrna lewini* .” Endangered Species Research 9: 221–228. 10.3354/esr00241.

[ece373261-bib-0017] Coelho, R. , F. Arocha , J. Báez , et al. 2025. “Revision of the Shortfin Mako Shark Size Distribution in the Atlantic.” 82: 1–22. 10.7916/D8S2ZD1B.

[ece373261-bib-0018] Colloca, F. , G. Scarcella , and S. Libralato . 2017. “Recent Trends and Impacts of Fisheries Exploitation on Mediterranean Stocks and Ecosystems.” Frontiers in Marine Science 4: 244. 10.3389/fmars.2017.00244.

[ece373261-bib-0110] Combosch, D. J. , S. Lemer , P. D. Ward , N. H. Landman , and G. Giribet . 2017. “Genomic Signatures of Evolution in Nautilus —An Endangered Living Fossil.” Molecular Ecology 26: 5923–5938. 10.1111/mec.14344. 28872211

[ece373261-bib-0019] Corrigan, S. , A. D. Lowther , L. B. Beheregaray , et al. 2018. “Population Connectivity of the Highly Migratory Shortfin Mako (*Isurus oxyrinchus* Rafinesque 1810) and Implications for Management in the Southern Hemisphere.” Frontiers in Ecology and Evolution 6: 187. 10.3389/fevo.2018.00187.

[ece373261-bib-0020] Danecek, P. , J. K. Bonfield , J. Liddle , et al. 2021. “Twelve Years of SAMtools and BCFtools.” GigaScience 10: giab008. 10.1093/gigascience/giab008. 33590861 PMC7931819

[ece373261-bib-0021] De Queiroz, K. 2007. “Species Concepts and Species Delimitation.” Systematic Biology 56: 879–886. 10.1080/10635150701701083. 18027281

[ece373261-bib-0022] Dedman, S. , J. H. Moxley , Y. P. Papastamatiou , et al. 2024. “Ecological Roles and Importance of Sharks in the Anthropocene Ocean.” Science 385: adl2362. 10.1126/science.adl2362. 39088608

[ece373261-bib-0023] Demirel, N. , M. Zengin , and A. Ulman . 2020. “First Large‐Scale Eastern Mediterranean and Black Sea Stock Assessment Reveals a Dramatic Decline.” Frontiers in Marine Science 7: 103. 10.3389/fmars.2020.00103.

[ece373261-bib-0024] Domingues, R. R. , I. V. Bunholi , D. Pinhal , A. Antunes , and F. F. Mendonça . 2021. “From Molecule to Conservation: DNA‐Based Methods to Overcome Frontiers in the Shark and Ray Fin Trade.” Conservation Genetics Resources 13: 231–247. 10.1007/s12686-021-01194-8.

[ece373261-bib-0025] Domingues, R. R. , A. W. S. Hilsdorf , and O. B. F. Gadig . 2018. “The Importance of Considering Genetic Diversity in Shark and Ray Conservation Policies.” Conservation Genetics 19: 501–525. 10.1007/s10592-017-1038-3.

[ece373261-bib-0026] Domingues, R. R. , V. A. Mastrochirico‐Filho , N. J. Mendes , et al. 2022. “Gene‐Associated Markers as a Genomic and Transcriptomic Resource for a Highly Migratory and Apex Predator Shark (*Isurus oxyrinchus*).” Marine Biology 169: 109. 10.1007/s00227-022-04094-z.

[ece373261-bib-0027] Dulvy, N. K. , D. J. Allen , G. M. Ralph , and R. H. L. Walls . 2016. The Conservation Status of Sharks, Rays and Chimaeras in the Mediterranean Sea. Vol. 14. IUCN.

[ece373261-bib-0028] Dulvy, N. K. , J. K. Baum , S. Clarke , et al. 2008. “You Can Swim but You Can't Hide: The Global Status and Conservation of Oceanic Pelagic Sharks and Rays.” Aquatic Conservation: Marine and Freshwater Ecosystems 18: 459–482. 10.1002/aqc.975.

[ece373261-bib-0029] Dulvy, N. K. , N. Pacoureau , C. L. Rigby , et al. 2021. “Overfishing Drives Over One‐Third of All Sharks and Rays Toward a Global Extinction Crisis.” Current Biology 31: 4773–4787. 10.1016/j.cub.2021.08.062. 34492229

[ece373261-bib-0030] Dulvy, N. K. , C. A. Simpfendorfer , L. N. K. Davidson , et al. 2017. “Challenges and Priorities in Shark and Ray Conservation.” Current Biology 27: R565–R572. 10.1016/j.cub.2017.04.038. 28586694

[ece373261-bib-0031] Estes, J. A. , M. Heithaus , D. J. McCauley , D. B. Rasher , and B. Worm . 2016. “Megafaunal Impacts on Structure and Function of Ocean Ecosystems.” Annual Review of Environment and Resources 41: 83–116. 10.1146/annurev-environ-110615-085622.

[ece373261-bib-0032] European Commission, Joint Research Centre, Scientific, Technical and Economic Committee for Fisheries . 2025. “Methodologies for Mediterranean Stock Assessments (STECF‐25‐01).” Scientific, technical and economic committee for fisheries STECF‐25‐01. Publications Office of the European Union, Luxembourg. 10.2760/1568886.

[ece373261-bib-0033] Excoffier, L. , and H. E. Lischer . 2010. “Arlequin Suite Ver 3.5: A New Series of Programs to Perform Population Genetics Analyses Under Linux and Windows.” Molecular Ecology Resources 10: 564–567. 21565059 10.1111/j.1755-0998.2010.02847.x

[ece373261-bib-0034] Falush, D. , M. Stephens , and J. K. Pritchard . 2003. “Inference of Population Structure Using Multilocus Genotype Data: Linked Loci and Correlated Allele Frequencies.” Genetics 164: 1567–1587. 12930761 10.1093/genetics/164.4.1567 PMC1462648

[ece373261-bib-0035] Fernández‐Corredor, E. , J. Ouled‐Cheikh , J. Navarro , and M. Coll . 2024. “An Overview of the Ecological Roles of Mediterranean Chondrichthyans Through Extinction Scenarios.” Reviews in Fish Biology and Fisheries 34: 421–438. 10.1007/s11160-023-09822-2.

[ece373261-bib-0036] Ferretti, F. , R. A. Myers , F. Serena , and H. K. Lotze . 2008. “Loss of Large Predatory Sharks From the Mediterranean Sea.” Conservation Biology 22: 952–964. 10.1111/j.1523-1739.2008.00938.x. 18544092

[ece373261-bib-0037] Ferretti, F. , B. Worm , G. L. Britten , M. R. Heithaus , and H. K. Lotze . 2010. “Patterns and Ecosystem Consequences of Shark Declines in the Ocean.” Ecology Letters 13: 1055–1071. 10.1111/j.1461-0248.2010.01489.x. 20528897

[ece373261-bib-0038] Foll, M. , and O. Gaggiotti . 2008. “A Genome‐Scan Method to Identify Selected Loci Appropriate for Both Dominant and Codominant Markers: A Bayesian Perspective.” Genetics 180: 977–993. 18780740 10.1534/genetics.108.092221 PMC2567396

[ece373261-bib-0039] Fowler, S. , A. Bräutigam , N. Okes , and G. Sant . 2021. “Conservation, Fisheries, Trade and Management Status of CITES‐Listed Sharks.”

[ece373261-bib-0040] Francis, M. P. , M. S. Shivji , C. A. J. Duffy , et al. 2018. “Oceanic Nomad or Coastal Resident? Behavioural Switching in the Shortfin Mako Shark (*Isurus oxyrinchus*).” Marine Biology 166: 5. 10.1007/s00227-018-3453-5.

[ece373261-bib-0041] Gallagher, A. J. , E. S. Orbesen , N. Hammerschlag , and J. E. Serafy . 2014. “Vulnerability of Oceanic Sharks as Pelagic Longline Bycatch.” Global Ecology and Conservation 1: 50–59. 10.1016/j.gecco.2014.06.003.

[ece373261-bib-0042] Gibson Banks, K. , D. M. Coffey , M. R. Fisher , and G. W. Stunz . 2025. “To Stay or Go: Movement, Behavior, and Habitat Use of Shortfin Mako Sharks ( *Isurus oxyrinchus* ) in the Gulf of Mexico.” Frontiers in Marine Science 12: 1562581. 10.3389/fmars.2025.1562581.

[ece373261-bib-0043] Gilman, E. , M. Chaloupka , L. R. Benaka , et al. 2022. “Phylogeny Explains Capture Mortality of Sharks and Rays in Pelagic Longline Fisheries: A Global Meta‐Analytic Synthesis.” Scientific Reports 12: 18164. 10.1038/s41598-022-21976-w. 36307432 PMC9616952

[ece373261-bib-0044] González, M. T. , N. V. Leiva , P. M. Zárate , and J. A. Baeza . 2023. “Regional (South‐Eastern Pacific Ocean) Population Genetics and Global Phylogeography of Two Endangered Highly Migratory Pelagic Sharks, the Blue Shark *Prionace glauca* and Shortfin Mako * isurus oxyrinchus * .” Aquatic Conservation: Marine and Freshwater Ecosystems 33: 1098–1115. 10.1002/aqc.3987.

[ece373261-bib-0045] Hedgecock, D. , P. H. Barber , and S. Edmands . 2007. “Genetic Approaches to Measuring Connectivity.” Oceanography 20: 70–79.

[ece373261-bib-0046] Heist, E. J. , J. A. Musick , and J. E. Graves . 1996. “Genetic Population Structure of the Shortfin Mako ( *Isurus oxyrinchus* ) Inferred From Restriction Fragment Length Polymorphism Analysis of Mitochondrial DNA.” Canadian Journal of Fisheries and Aquatic Sciences 53: 583–588. 10.1139/f95-245.

[ece373261-bib-0047] ICCAT . 2019. “Report of the 2019 Shortfin Mako Shark Stock Assessment Update Meeting.” SMA SHK SA INTERSESSIONAL MEETING. ICCAT, Madrid.

[ece373261-bib-0048] ICCAT . 2021. “Recommendation by ICCAT on the Conservation of the North Atlantic Stock of Shortfin Mako Caught in Association With ICCAT Fisheries.”

[ece373261-bib-0049] ICCAT . 2025. “Report of the 2025 ICCAT Shortfin Mako Shark Data Preparatory Meeting.” Malaga. pacodulvy.

[ece373261-bib-0050] Jombart, T. , and I. Ahmed . 2011. “Adegenet 1.3‐1: New Tools for the Analysis of Genome‐Wide SNP Data.” Bioinformatics 27: 3070–3071. 10.1093/bioinformatics/btr521. 21926124 PMC3198581

[ece373261-bib-0051] Jombart, T. , S. Devillard , and F. Balloux . 2010. “Discriminant Analysis of Principal Components: A New Method for the Analysis of Genetically Structured Populations.” BMC Genetics 11: 94. 20950446 10.1186/1471-2156-11-94 PMC2973851

[ece373261-bib-0052] Kacev, D. 2015. “Understanding Population Connectivity in Shortfin Mako Shark ( *Isurus oxyrinchus* ) at Multiple Spatial Scales.” PhD, University of California Davis.

[ece373261-bib-0053] Kai, M. 2021. “Are the Current IUCN Category and CITES Listing Appropriate for the Conservation and Management of Shortfin Mako, *Isurus oxyrinchus* , in the North Pacific Ocean?” Marine Policy 134: 104790. 10.1016/j.marpol.2021.104790.

[ece373261-bib-0054] Kool, J. T. , A. Moilanen , and E. A. Treml . 2013. “Population Connectivity: Recent Advances and New Perspectives.” Landscape Ecology 28: 165–185. 10.1007/s10980-012-9819-z.

[ece373261-bib-0055] Kopelman, N. M. , J. Mayzel , M. Jakobsson , N. A. Rosenberg , and I. Mayrose . 2015. “Clumpak: A Program for Identifying Clustering Modes and Packaging Population Structure Inferences Across K.” Molecular Ecology Resources 15: 1179–1191. 25684545 10.1111/1755-0998.12387 PMC4534335

[ece373261-bib-0057] Lazaris, A. , D. V. Politikos , E. Tzanatos , and V. Vassilopoulou . 2025. “Disentangling the Sustainability of Longline Demersal Fisheries in Central Mediterranean.” Reviews in Fish Biology and Fisheries 36: 25. 10.1007/s11160-025-10028-x.

[ece373261-bib-0058] Leone, A. , S. Arnaud‐Haond , M. Babbucci , et al. 2024. “Population Genomics of the Blue Shark, *Prionace glauca* , Reveals Different Populations in the Mediterranean Sea and the Northeast Atlantic.” Evolutionary Applications 17: e70005. 10.1111/eva.70005. 39296540 PMC11408569

[ece373261-bib-0059] Lewison, R. , L. Crowder , A. Read , and S. Freeman . 2004. “Understanding Impacts of Fisheries Bycatch on Marine Megafauna.” Trends in Ecology & Evolution 19: 598–604. 10.1016/j.tree.2004.09.004.

[ece373261-bib-0060] Li, H. 2013. “Aligning Sequence Reads, Clone Sequences and Assembly Contigs With BWA‐MEM.” 10.48550/arXiv.1303.3997.

[ece373261-bib-0061] Liu, X. , and Y.‐X. Fu . 2020. “Stairway Plot 2: Demographic History Inference With Folded SNP Frequency Spectra.” Genome Biology 21: 280. 10.1186/s13059-020-02196-9. 33203475 PMC7670622

[ece373261-bib-0062] Lowe, W. H. , and F. W. Allendorf . 2010. “What Can Genetics Tell Us About Population Connectivity?” Molecular Ecology 19: 3038–3051. 10.1111/j.1365-294X.2010.04688.x. 20618697

[ece373261-bib-0063] Maia, A. , N. Queiroz , H. N. Cabral , A. M. Santos , and J. P. Correia . 2007. “Reproductive Biology and Population Dynamics of the Shortfin Mako, *Isurus oxyrinchus* Rafinesque, 1810, Off the Southwest Portuguese Coast, Eastern North Atlantic.” Journal of Applied Ichthyology 23: 246–251. 10.1111/j.1439-0426.2007.00849.x.

[ece373261-bib-0064] Malinsky, M. , E. Trucchi , D. J. Lawson , and D. Falush . 2018. “RADpainter and fineRADstructure: Population Inference From RADseq Data.” Molecular Biology and Evolution 35: 1284–1290. 29474601 10.1093/molbev/msy023 PMC5913677

[ece373261-bib-0065] Mantel, N. 1967. “The Detection of Disease Clustering and a Generalized Regression Approach.” Cancer Research 27: 209–220. 6018555

[ece373261-bib-0066] Marsaglia, L. , A. Parisi , S. Libralato , et al. 2025. “Shedding Light on Trawl Fishing Activity in the Mediterranean Sea With Remote Sensing Data.” ICES Journal of Marine Science 82: fsae153. 10.1093/icesjms/fsae153.

[ece373261-bib-0067] McLaughlin, J. F. , and K. Winker . 2020. “An Empirical Examination of Sample Size Effects on Population Demographic Estimates in Birds Using Single Nucleotide Polymorphism (SNP) Data.” PeerJ 8: e9939. 10.7717/peerj.9939. 32995092 PMC7501783

[ece373261-bib-0068] Medeiros, B. A. S. d. , and B. D. Farrell . 2018. “Whole‐Genome Amplification in Double‐Digest RADseq Results in Adequate Libraries but Fewer Sequenced Loci.” PeerJ 6: e5089. 10.7717/peerj.5089. 30038852 PMC6054070

[ece373261-bib-0069] Mehlrose, M. 2022. “Determination of Atlantic Shortfin Mako Shark ( *Isurus oxyrinchus* ) Population Genetic Structure and Comparison of Mitogenomic Markers.” All HCAS Student Capstones, Theses, and Dissertations. https://nsuworks.nova.edu/hcas_etd_all/107.

[ece373261-bib-0070] Milazzo, M. , C. Cattano , S. A. A. Al Mabruk , and I. Giovos . 2021. “Mediterranean Sharks and Rays Need Action.” Science Letters 371: 355–356. 10.1126/science.abg1943 33479144

[ece373261-bib-0071] Moro, S. , S. Valente , M. Arcioni , et al. 2025. “Living on the Extinction Edge: Resilience to Fishing and Rebound Potential of the Mediterranean Elasmobranchs.” Fish and Fisheries 26: 772–789. 10.1111/faf.12911.

[ece373261-bib-0072] Moses, K. , L. Katsis , P. Griffith , R. Kemp , and A. Dancer . 2022. An Introduction to Satellite Technologies for Tracking Wildlife. Zoological Society of London.

[ece373261-bib-0073] Mucientes, G. , A. Alonso‐Fernández , M. Vedor , D. W. Sims , and N. Queiroz . 2025. “Discovery of a Potential Open Ocean Nursery for the Endangered Shortfin Mako Shark in a Global Fishing Hotspot.” Scientific Reports 15: 2190. 10.1038/s41598-025-85572-4. 39820057 PMC11739380

[ece373261-bib-0074] Natanson, L. J. , N. E. Kohler , D. Ardizzone , G. M. Cailliet , S. P. Wintner , and H. F. Mollet . 2006. “Validated Age and Growth Estimates for the Shortfin Mako, *Isurus oxyrinchus* , in the North Atlantic Ocean.” Environmental Biology of Fishes 77: 367–383. 10.1007/s10641-006-9127-z.

[ece373261-bib-0075] Natanson, L. J. , M. Winton , H. Bowlby , et al. 2020. “Updated Reproductive Parameters for the Shortfin Mako ( *Isurus oxyrinchus* ) in the North Atlantic Ocean With Inferences of Distribution by Sex and Reproductive Stage.” Fishery Bulletin 118: 21–36. 10.7755/FB.118.1.3.

[ece373261-bib-0076] Nazareno, A. G. , J. B. Bemmels , C. W. Dick , and L. G. Lohmann . 2017. “Minimum Sample Sizes for Population Genomics: An Empirical Study From an Amazonian Plant Species.” Molecular Ecology Resources 17: 1136–1147. 10.1111/1755-0998.12654. 28078808

[ece373261-bib-0077] Olsen, M. T. , L. W. Andersen , R. Dietz , J. Teilmann , T. Härkönen , and H. R. Siegismund . 2014. “Integrating Genetic Data and Population Viability Analyses for the Identification of Harbour Seal ( *Phoca vitulina* ) Populations and Management Units.” Molecular Ecology 23: 815–831. 10.1111/mec.12644. 24382213

[ece373261-bib-0078] Pacoureau, N. , J. K. Carlson , H. K. Kindsvater , et al. 2023. “Conservation Successes and Challenges for Wide‐Ranging Sharks and Rays.” Proceedings of the National Academy of Sciences 120: e2216891120. 10.1073/pnas.2216891120. PMC9945978 36689654

[ece373261-bib-0079] Pacoureau, N. , C. L. Rigby , P. M. Kyne , et al. 2021. “Half a Century of Global Decline in Oceanic Sharks and Rays.” Nature 589: 567–571. 10.1038/s41586-020-03173-9. 33505035

[ece373261-bib-0080] Palumbi, S. R. 1994. “Genetic Divergence, Reproductive Isolation, and Marine Speciation.” Annual Review of Ecology and Systematics 25: 547–572.

[ece373261-bib-0081] Papaconstantinou, C. , and H. Farrugio . 2000. “Fisheries in the Mediterranean.” Mediterranean Marine Science 1: 5–18. 10.12681/mms.2.

[ece373261-bib-0082] Paris, J. R. , J. R. Stevens , and J. M. Catchen . 2017. “Lost in Parameter Space: A Road Map for Stacks.” Methods in Ecology and Evolution 8: 1360–1373. 10.1111/2041-210X.12775.

[ece373261-bib-0083] Pascual, M. , B. Rives , C. Schunter , and E. Macpherson . 2017. “Impact of Life History Traits on Gene Flow: A Multispecies Systematic Review Across Oceanographic Barriers in the Mediterranean Sea Ed T‐Y Chiang.” PLoS One 12: e0176419. 10.1371/journal.pone.0176419. 28489878 PMC5425013

[ece373261-bib-0084] Patarnello, T. , F. A. M. J. Volckaert , and R. Castilho . 2007. “Pillars of Hercules: Is the Atlantic–Mediterranean Transition a Phylogeographical Break?” Molecular Ecology 16: 4426–4444. 10.1111/j.1365-294X.2007.03477.x. 17908222

[ece373261-bib-0085] Pembleton, L. , N. Cogan , and J. Forster . 2013. “StAMPP: An R Package for Calculation of Genetic Differentiation and Structure of Mixed‐Ploidy Level Populations.” Molecular Ecology Resources 13: 946–952. 10.1111/1755-0998.12129. 23738873

[ece373261-bib-0086] Peterson, B. K. , J. N. Weber , E. H. Kay , H. S. Fisher , and H. E. Hoekstra . 2012. “Double Digest RADseq: An Inexpensive Method for De Novo SNP Discovery and Genotyping in Model and Non‐Model Species Ed L Orlando.” PLoS One 7: e37135. 10.1371/journal.pone.0037135. 22675423 PMC3365034

[ece373261-bib-0087] Prato, G. , P. Guidetti , F. Bartolini , L. Mangialajo , and P. Francour . 2013. “The Importance of High‐Level Predators in Marine Protected Area Management: Consequences of Their Decline and Their Potential Recovery in the Mediterranean Context.” Advances in Oceanography and Limnology 4: 176–193. 10.1080/19475721.2013.841754.

[ece373261-bib-0088] Pritchard, J. K. , M. Stephens , and P. Donnelly . 2000. “Inference of Population Structure Using Multilocus Genotype Data.” Genetics 155: 945–959. 10.1093/genetics/155.2.945. 10835412 PMC1461096

[ece373261-bib-0089] Queiroz, N. , N. E. Humphries , A. Couto , et al. 2019. “Global Spatial Risk Assessment of Sharks Under the Footprint of Fisheries.” Nature 572: 461–466. 10.1038/s41586-019-1444-4. 31340216

[ece373261-bib-0090] Reuschel, S. , J. A. Cuesta , and C. D. Schubart . 2010. “Marine Biogeographic Boundaries and Human Introduction Along the European Coast Revealed by Phylogeography of the Prawn *Palaemon elegans* .” Molecular Phylogenetics and Evolution 55: 765–775. 10.1016/j.ympev.2010.03.021. 20307676

[ece373261-bib-0091] Rigby, C. L. , R. Barreto , J. Carlson , et al. 2019. “ *Isurus oxyrinchus* .” The IUCN Red List of Threatened Species. 10.2305/IUCN.UK.2019-1.RLTS.T39341A2903170.en.

[ece373261-bib-0092] Rousset, F. 1997. “Genetic Differentiation and Estimation of Gene Flow From F‐Statistics Under Isolation by Distance.” Genetics 145: 1219–1228. 9093870 10.1093/genetics/145.4.1219 PMC1207888

[ece373261-bib-0093] Santos, C. C. , A. Domingo , J. Carlson , et al. 2021. “Movements, Habitat Use, and Diving Behavior of Shortfin Mako in the Atlantic Ocean.” Frontiers in Marine Science 8: 686343. 10.3389/fmars.2021.686343.

[ece373261-bib-0094] Scacco, U. , E. Gennari , S. Di Crescenzo , and E. Fanelli . 2023. “Looking Into the Prevalence of Bycatch Juveniles of Critically Endangered Elasmobranchs: A Case Study From Pelagic Longline and Trammel Net Fisheries of the Asinara Gulf (Western Mediterranean).” Frontiers in Marine Science 10: 1303961. 10.3389/fmars.2023.1303961.

[ece373261-bib-0095] Schrey, A. W. , and E. J. Heist . 2003. “Microsatellite Analysis of Population Structure in the Shortfin Mako ( *Isurus oxyrinchus* ).” Canadian Journal of Fisheries and Aquatic Sciences 60: 670–675. 10.1139/f03-064.

[ece373261-bib-0096] Shea, B. D. , T. K. Chapple , K. Echwikhi , et al. 2024. “First Satellite Track of a Juvenile Shortfin Mako Shark ( *Isurus oxyrinchus* ) in the Mediterranean Sea.” Frontiers in Marine Science 11: 1423507. 10.3389/fmars.2024.1423507.

[ece373261-bib-0097] Sims, D. W. , G. Mucientes , and N. Queiroz . 2021. “Shortfin Mako Sharks Speeding to the Brink Ed J Sills.” Science 371: 355. 10.1126/science.abg2355. 33479143

[ece373261-bib-0098] Six, K. D. , U. Mikolajewicz , and G. Schmiedl . 2024. “Modelling Mediterranean Ocean Biogeochemistry of the Last Glacial Maximum.” Climate of the Past 20: 1785–1816. 10.5194/cp-20-1785-2024.

[ece373261-bib-0099] Spaet, J. L. Y. , R. W. Jabado , A. C. Henderson , A. B. M. Moore , and M. L. Berumen . 2015. “Population Genetics of Four Heavily Exploited Shark Species Around the Arabian Peninsula.” Ecology and Evolution 5: 2317–2332. 10.1002/ece3.1515. 26120422 PMC4475365

[ece373261-bib-0100] Stanhope, M. J. , K. M. Ceres , Q. Sun , et al. 2023. “Genomes of Endangered Great Hammerhead and Shortfin Mako Sharks Reveal Historic Population Declines and High Levels of Inbreeding in Great Hammerhead.” iScience 26: 105815. 10.1016/j.isci.2022.105815. 36632067 PMC9826928

[ece373261-bib-0101] Stoffel, M. A. , E. Humble , A. J. Paijmans , et al. 2018. “Demographic Histories and Genetic Diversity Across Pinnipeds Are Shaped by Human Exploitation, Ecology and Life‐History.” Nature Communications 9: 4836. 10.1038/s41467-018-06695-z. PMC6240053 30446730

[ece373261-bib-0102] Taguchi, M. , T. Kitamura , and K. Yokawa . 2011. “Genetic Population Structure of Shortfin Mako ( *Isurus oxyrinchus* ) Inferred From Mitochondrial DNA on Inter‐ Oceanic Scale. In ‘International Scientific Commitee for Tuna and Tuna‐Like Species in the North Pacific Ocean’.”

[ece373261-bib-0103] Taguchi, M. , Y. Shigenobu , M. Ohkubo , et al. 2013. “Characterization of 12 Polymorphic Microsatellite DNA Loci in the Blue Shark, *Prionace glauca* , Isolated by Next Generation Sequencing Approach.” Conservation Genetics Resources 5: 117–119. 10.1007/s12686-012-9746-y.

[ece373261-bib-0104] Toews, D. P. L. , and A. Brelsford . 2012. “The Biogeography of Mitochondrial and Nuclear Discordance in Animals.” Molecular Ecology 21: 3907–3930. 10.1111/j.1365-294X.2012.05664.x. 22738314

[ece373261-bib-0105] Vella, N. , and A. Vella . 2023. “Phylogeographic Analyses of the Shortfin Mako, *Isurus oxyrinchus* Rafinesque, 1810 (Chondrichthyes: Lamniformes) From the Central Mediterranean Sea, a Critically Endangered Species in the Region.” Fishes 8: 520. 10.3390/fishes8100520.

[ece373261-bib-0106] Veríssimo, A. , Í. Sampaio , J. R. McDowell , et al. 2017. “World Without Borders—Genetic Population Structure of a Highly Migratory Marine Predator, the Blue Shark ( *Prionace glauca* ).” Ecology and Evolution 7: 4768–4781. 10.1002/ece3.2987. 28690806 PMC5496551

[ece373261-bib-0107] Wright, S. 1943. “Isolation by Distance.” Genetics 28: 114–138. 17247074 10.1093/genetics/28.2.114 PMC1209196

[ece373261-bib-0108] Yokoi, H. , H. Ijima , S. Ohshimo , and K. Yokawa . 2017. “Impact of Biology Knowledge on the Conservation and Management of Large Pelagic Sharks.” Scientific Reports 7: 10619. 10.1038/s41598-017-09427-3. 28878365 PMC5587552

[ece373261-bib-0109] Zheng, X. , D. Levine , J. Shen , S. M. Gogarten , C. Laurie , and B. S. Weir . 2012. “A High‐Performance Computing Toolset for Relatedness and Principal Component Analysis of SNP Data.” Bioinformatics 28: 3326–3328. 10.1093/bioinformatics/bts606. 23060615 PMC3519454

