# Supplementary material for: Evidence of Genetic Continuity in the Shortfin Mako Shark (Isurus oxyrinchus) Between the Eastern Atlantic and Mediterranean Sea
Source: Ecol Evol. 2026 Apr 6;16(4):e73261. doi: 10.1002/ece3.73261 (PMC13053171; doi:10.1002/ece3.73261)
Supplement: Supplementary file 1 — Data S1: ece373261‐sup‐0001‐Supinfo.pdf. [file ECE3-16-e73261-s001.pdf]

Supplementary Material

Evidence of genetic continuity in the shortfin mako shark (*Isurus oxyrinchus*) between the Eastern Atlantic and Mediterranean Sea

Abridged title: Genetic connectivity of shortfin mako shark

Authors: Gambardella Chiara[1,2]*, Giannelli Francesco[1,3,4], Fernandez-Corredor Elena[5], García-Barcelona Salvador[6], Jenrette Jeremy[7], Moro Stefano[8], Shea Brendan[7], Colloca Francesco[8], Romeo Teresa[2,9], Echwiki Khaled[10], Zammit-Chatti Maissa[11], Lemsi Chiheb[12,13], Ferretti Francesco[7], Trucchi Emiliano[1], Taboada Sergio[14], Navarro Joan[5]

[1] Università Politecnica delle Marche, Life and Environmental Sciences, Via Brecce Bianche, snc, 60131 Ancona, Ancona, IT

[2] Stazione Zoologica Anton Dohrn Napoli, Sicily Marine Center- BEOM Department, Contrada Porticatello, 29 98167 Messina, Sicily, IT

[3] Human Evolution Program, Department of Organismal Biology, Evolutionary Biology Centre, Uppsala University, Uppsala, Swedend

[4] Center for the Human Past, Department of Organismal Biology, Uppsala University, Uppsala, Sweden

[5] Institut de Ciències del Mar (ICM), CSIC, Pg. Marítim de la Barceloneta, 37, 08003 Barcelona, ES

[6] Centro Oceanográfico de Málaga, Instituto Español de Oceanografía (IEO-CSIC), Puerto de Málaga, 29002 Málaga, ES

[7] Virginia Tech, Department of Fish and Wildlife Conservation, Blacksburg, 24061 VA, US

[8] Stazione Zoologica Anton Dohrn, Department of Integrative Marine Ecology, Via Gregorio Allegri 1, 00198, Rome, Lazio, IT

[9] Italian Institute for Environmental Protection and Research, ISPRA, 00144 Rome, Italy

[10] University of Gabes, Laboratory of Ecology and Environment: LR24ES17, 6029 Gabes, TN

[11] University of Jendouba, Higher Institute of Biotechnology of Beja, 9000 Jendouba, TN

[12] University of Sfax, Faculty of Sciences, 3029 Sfax, TN

[13] Department of Earth and Marine Sciences (DiSTeM), University of Palermo, Palermo, Italy

[14] Departamento de Biodiversidad y Biología Evolutiva, Museo Nacional de Ciencias Naturales (MNCN), CSIC, Madrid, ES

***Corresponding author:** c.gambardella@pm.univpm.it

Table S 1 Metadata of the samples collected. Code: the unique code for each individual; Area: the area in which it was accidentally caught; Fork length: the fork length expressed in cm; Sex: the sex (male and female)

Code	Area	Fork Length (cm)	Sex
SIC24-7	Sicilian Channel		F
SIC24-8	Sicilian Channel	165.51	F
SIC23-5	Sicilian Channel	73.539	F
SIC24-6	Sicilian Channel		
SIC23-7	Sicilian Channel	63.32	F
SIC23-8	Sicilian Channel	230.54	M
SIC23-6	Sicilian Channel	65.178	F
EMS24-1	Eastern Mediterranean	109.77	M
SIC23-3	Sicilian Channel	74	F
SIC23-1	Sicilian Channel	75	F
SIC23-2	Sicilian Channel	79	M
TYR14-1	Tyrrhenian Sea		
TYR12-1	Tyrrhenian Sea		
TYR24-1	Tyrrhenian Sea	209.173	M
SIC17-1	Sicilian Channel	84.687	F
SIC22-1	Sicilian Channel	64.249	M
BAL17-1	Balearic Sea	160	
BAL17-2	Balearic Sea	87.5	
BAL17-3	Balearic Sea	87.5	
GOC17-13	Gulf of Cadiz	140	
GOC17-8	Gulf of Cadiz	125	
GOC17-18	Gulf of Cadiz	133	
GOC17-22	Gulf of Cadiz	136	
GOC17-4	Gulf of Cadiz	133	
GOC17-7	Gulf of Cadiz	172	
GOC17-20	Gulf of Cadiz	104	
GOC17-19	Gulf of Cadiz	152	
GOC17-14	Gulf of Cadiz	93	
GOC17-17	Gulf of Cadiz	101	
GOC17-3	Gulf of Cadiz	188	
GOC17-16	Gulf of Cadiz	98	
GOC17-15	Gulf of Cadiz	145	
GOC17-5	Gulf of Cadiz	111	
GOC17-12	Gulf of Cadiz	160	
GOC17-21	Gulf of Cadiz	102	
GOC17-1	Gulf of Cadiz	124	
GOC17-10	Gulf of Cadiz	102	
GOC17-9	Gulf of Cadiz	137	

GOC17-2	Gulf of Cadiz	145	
GOC17-6	Gulf of Cadiz	135	
CAN18-1	Canary Islands	67	
CAN18-2	Canary Islands	67	
GOC18-1	Gulf of Cadiz	72.2	
GOC18-2	Gulf of Cadiz	141	
GOC18-7	Gulf of Cadiz	142	
GOC18-3	Gulf of Cadiz	189	
GOC18-6	Gulf of Cadiz	95	
GOC18-9	Gulf of Cadiz	102	
GOC18-10	Gulf of Cadiz	143	F
GOC18-5	Gulf of Cadiz	135	F
GOC18-8	Gulf of Cadiz	106	
GOC18-4	Gulf of Cadiz	105	
GOC18-11	Gulf of Cadiz	149	M
GOC19-1	Gulf of Cadiz	127.58	
GOC19-3	Gulf of Cadiz	143.26	
GOC19-4	Gulf of Cadiz	138	
GOC19-2	Gulf of Cadiz	174	
CAN19-8	Canary Islands	120	
CAN19-9	Canary Islands	97	
CAN19-1	Canary Islands	116	
CAN19-5	Canary Islands	115	
CAN19-4	Canary Islands	126	
CAN19-6	Canary Islands	118	
CAN19-7	Canary Islands	156	
CAN19-2	Canary Islands	106	
CAN19-3	Canary Islands	104	
BAL23-1	Balearic Sea	140	
EMS19-1	Eastern Mediterranean	253.765	F
SIC24-2	Sicilian Channel		
SIC24-3	Sicilian Channel		
SIC24-10	Sicilian Channel	54.03	F
SIC24-11	Sicilian Channel	79.113	F
SIC24-12	Sicilian Channel	109.77	F
SIC24-13	Sicilian Channel	109.77	F
SIC23-4	Sicilian Channel	88.403	F
SIC24-5	Sicilian Channel		
SIC24-9	Sicilian Channel	130.208	F
SIC24-4	Sicilian Channel	72.61	F
SIC24-1	Sicilian Channel	54.03	F
EMS17-1	Eastern Mediterranean		

EMS17-2	Eastern Mediterranean		F

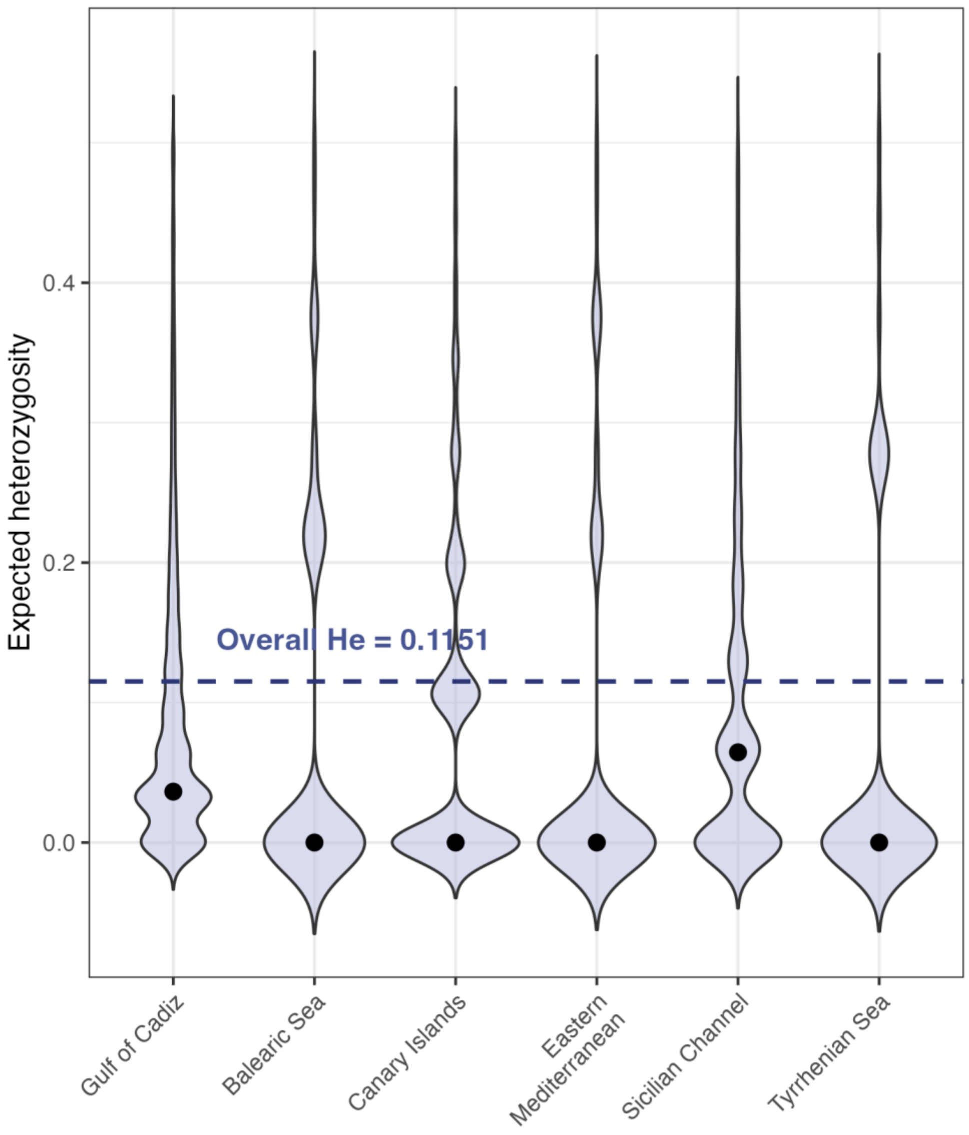

Figure S 1-Distribution of expected heterozygosity (He) across sampling regions of shortfin mako (Isurus oxyrinchus) in the Eastern Atlantic and Mediterranean Sea. Violin plots show the distribution of He per locus within each region, with black dots indicating median values. The dashed blue line represents the overall mean He across all regions (He = 0.1151)

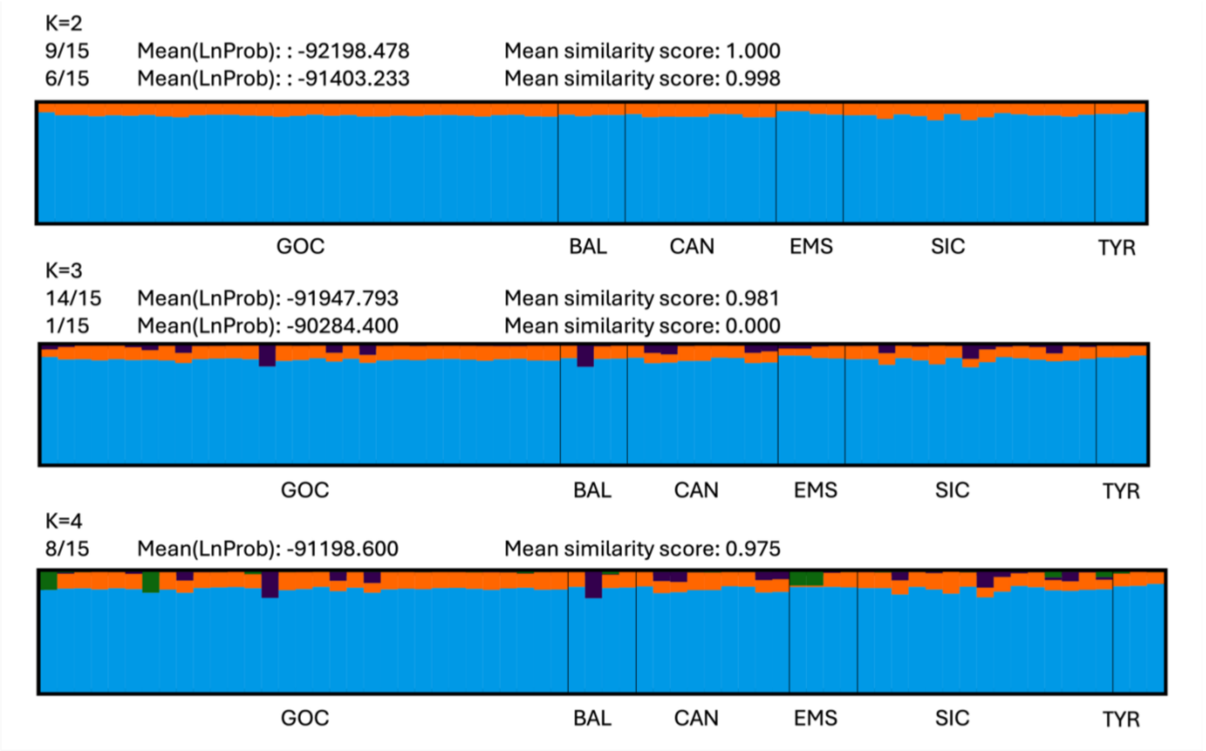

Figure S 2- Bayesian clustering analysis of shortfin mako sharks using STRUCTURE for K = 2, 3, and 4. Each vertical bar represents an individual, and colours indicate inferred ancestry proportions from the corresponding number of genetic clusters. Individuals are grouped by sampling region: Gulf of Cadiz (GOC), Balearic Sea (BAL), Canary Islands (CAN), Eastern Mediterranean (EMS), Sicilian Channel (SIC), and Tyrrhenian Sea (TYR). For each K, the mean log-likelihood of the data (Mean[LnProb]) and mean similarity scores across replicate runs are reported above the plots. While models with K > 1 identified artificial sub-clusters, these were not geographically consistent and did not alter the overall conclusion of panmixia (see main text).

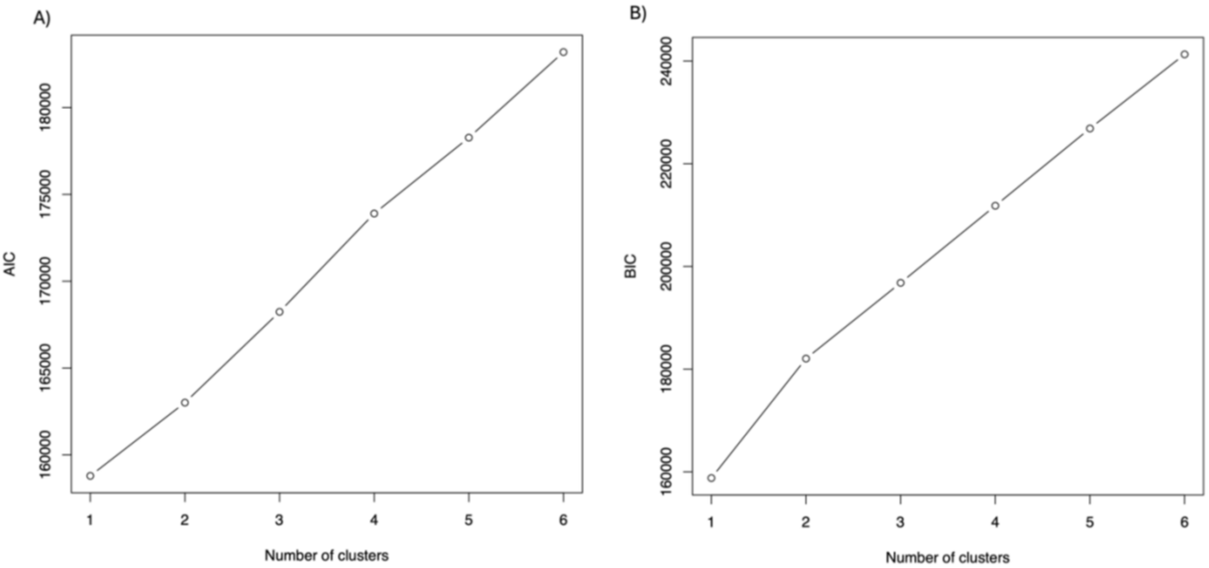

Figure S 3- Model selection plots for Discriminant Analysis of Principal Components (DAPC). (A) Akaike Information Criterion (AIC) and (B) Bayesian Information Criterion (BIC) values for models with 1–6 clusters. Both criteria identified K = 1 as the best-supported model.

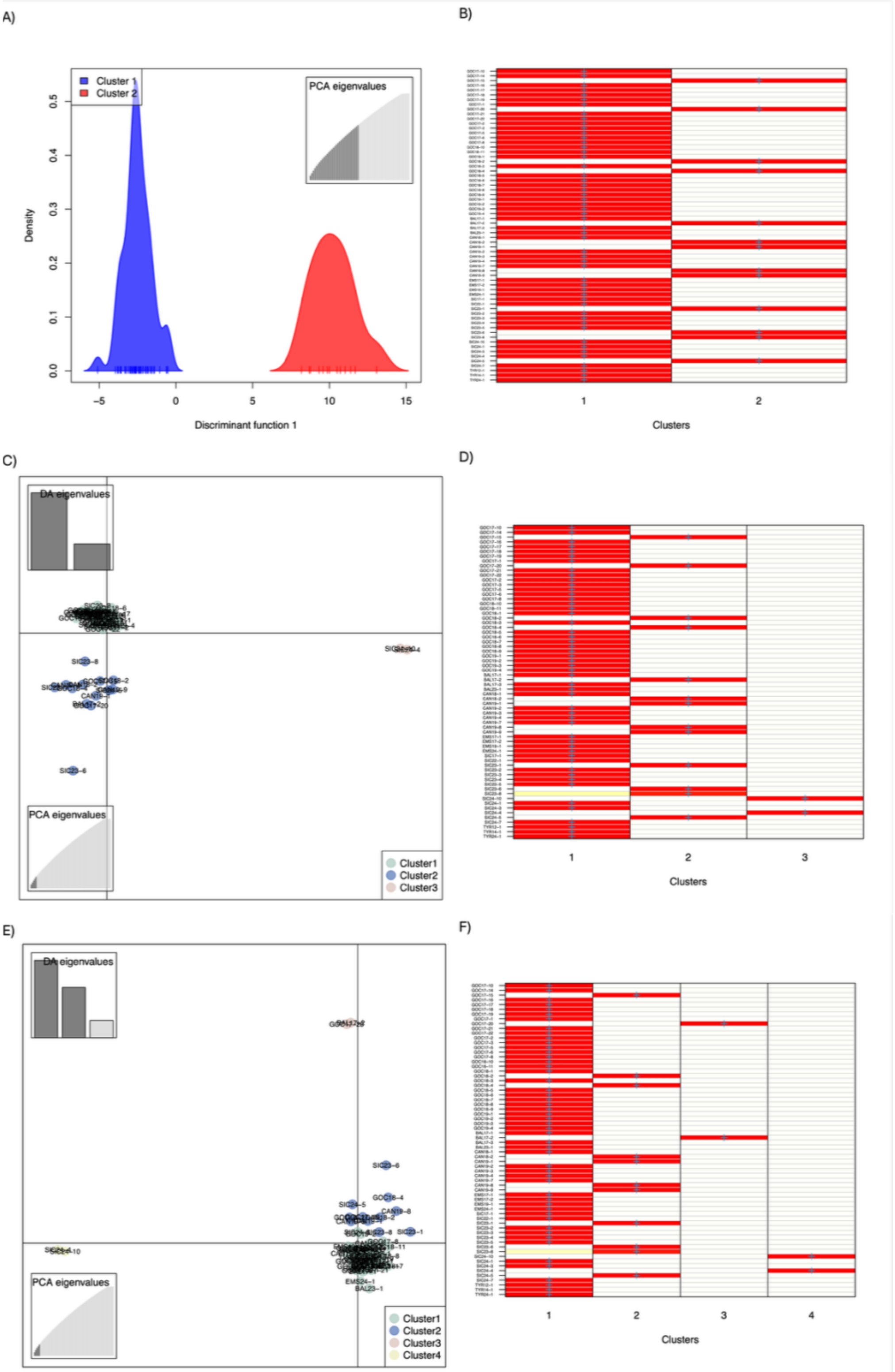

Figure S 4- Discriminant Analysis of Principal Components (DAPC) for shortfin mako sharks showing results for K = 2, 3, and 4 clusters. (A, C, E) Scatterplots of individuals projected onto discriminant functions, with colours representing inferred clusters. Insets show the proportion of variance explained by retained PCA eigenvalues (bottom left) and discriminant analysis eigenvalues (top left). (B, D, F) Assignment plots displaying individual membership probabilities for the corresponding number of clusters. Although forcing K = 2–4 identified artificial partitions, these clusters were not geographically consistent and individuals from different sampling regions overlapped extensively, supporting panmixia in the dataset.

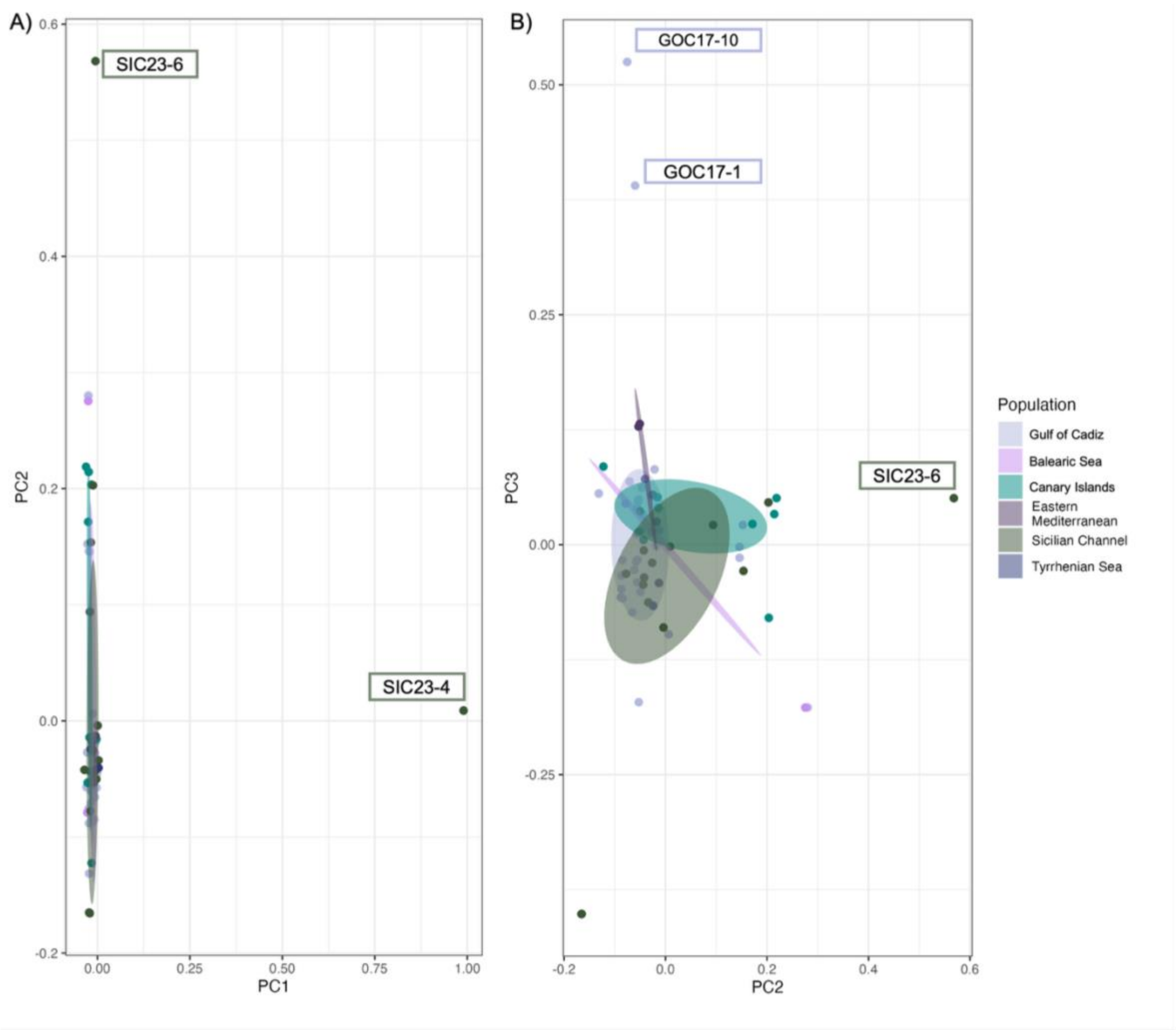

Figure S 5- Principal Component Analysis (PCA) of 66 shortfin mako sharks (4349 neutral SNPs). Individuals are represented as points coloured according to sampling region (Gulf of Cadiz in light violet, Balearic Sea in pink, Canary Islands in emerald, Eastern Mediterranean in violet, Sicilian Channel in dark green, and Tyrrhenian Sea in dark blue). Ellipses indicate 95% confidence intervals around group centroids. PC1 and PC2 explained 3.75% and 2.60% of the total genetic variance, respectively (6.35% combined). Ellipses indicate 95% confidence intervals around group centroids. PC2 and PC3 explained 2.60% and 2.20% of the total genetic variance, respectively (4.80% combined).

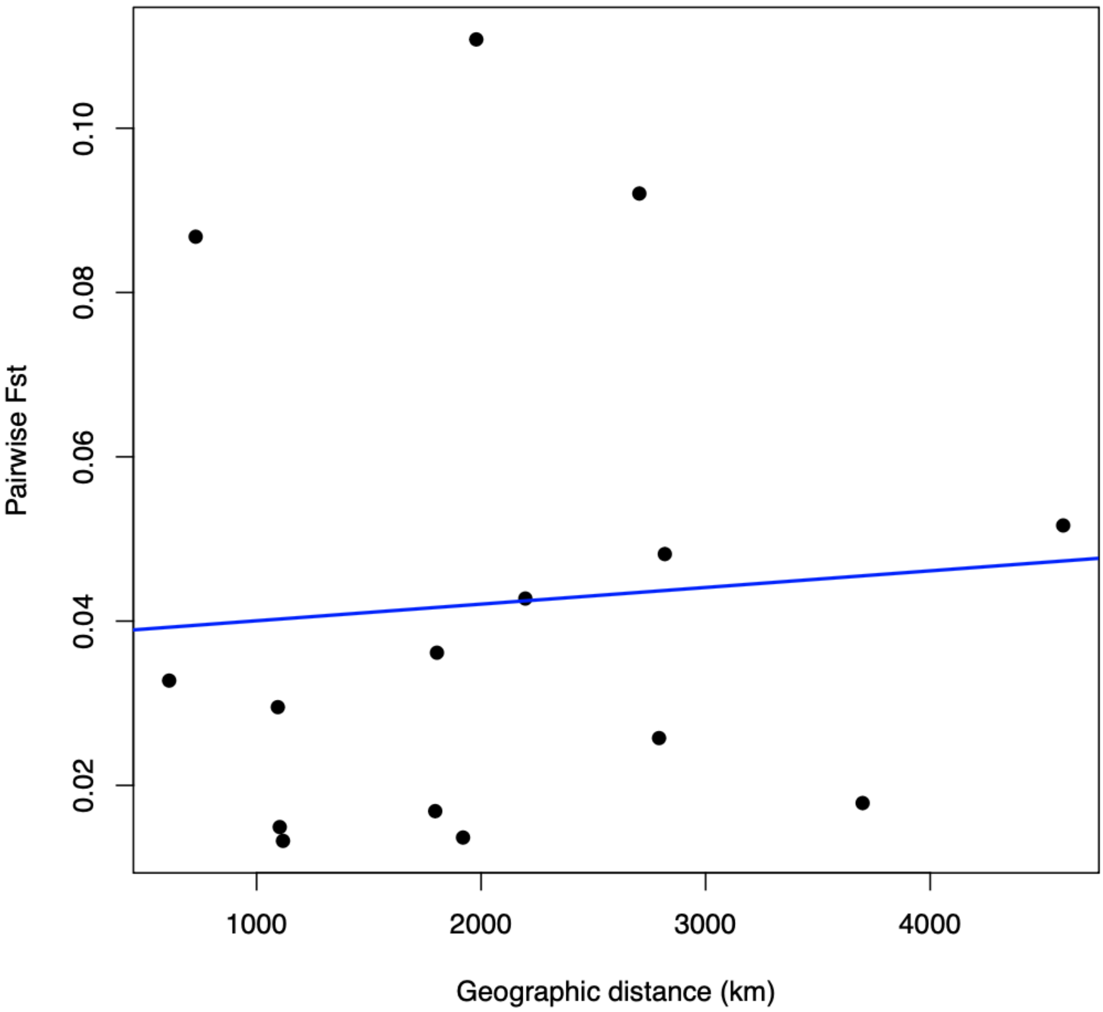

Figure S 6- Isolation by distance (IBD) analysis for shortfin mako sharks based on pairwise F_{ST} values plotted against geographic distance (km). The blue line represents the linear regression. The Mantel test revealed no significant correlation.between genetic and geographic distance ($p > 0.05$).

Mantel test Output:

Mantel statistic r: 0.07281
Significance: 0.40833
Upper quantiles of permutations (null model):

90%	95%	97.5%	99%
0.474	0.571	0.603	0.619

Permutation: free
Number of permutations: 719
